# Point-of-care Ratiometric Fluorescence Imaging of Tissue for the Diagnosis of Ovarian Cancer

**DOI:** 10.7150/thno.35322

**Published:** 2019-06-24

**Authors:** Xiaobo Zhou, Yawei Liu, Qiyu Liu, Luzhe Yan, Meng Xue, Wei Yuan, Mei Shi, Wei Feng, Congjian Xu, Fuyou Li

**Affiliations:** 1 Department of Chemistry & State Key Laboratory of Molecular Engineering of Polymers & Institute of Biomedicine Science, Fudan University, Shanghai 200433, China; 2 Department of Obstetrics and Gynecology of Shanghai Medical School & Shanghai Key Laboratory of Female Reproductive Endocrine Related Diseases & Obstetrics and Gynecology Hospital of Fudan University, Fudan University Shanghai 200011, China; 3 The High School Affiliated to Renmin University of China, Beijing 100080, China

**Keywords:** ovarian cancer detection, γ-glutamyltranspeptidase, point-of-care detection, ratiometric fluorescence imaging

## Abstract

During a minimally invasive tumor resection procedure, it is still a challenge to rapidly and accurately trace tiny malignant tumors in real time. Fluorescent molecular imaging is considered an efficient method of localizing tumors during surgery due to its high sensitivity and biosafety. On the basis of the fact that γ-glutamyltranspeptidase (GGT) is overexpressed in ovarian cancer, we herein designed a highly sensitive ratiometric fluorescent GGT-responsive probe Py-GSH for rapid tumor detection.

**Methods:** The GGT response probe (Py-GSH) was constructed by using GSH group as a response group and pyrionin B as a fluorescent reporter. Py-GSH was characterized for photophysical properties, response speed and selectivity of GGT and response mechanism. The anti-interference ability of ratiometric probe Py-GSH to probe concentration and excitation power was evaluated both *in vitro* and in tissue. The biocompatibility and toxicity of the ratiometric probe was examined using cytoxicity test. The GGT levels in different lines of cells were determined by ratiometric fluorescence imaging and cytometry analysis.

**Results:** The obtained probe capable to rapidly monitored GGT activity in aqueous solution with 170-fold ratio change. By ratiometric fluorescence imaging, the probe Py-GSH was also successfully used to detect high GGT activity in solid tumor tissues and small peritoneal metastatic tumors (~1 mm in diameter) in a mouse model. In particular, this probe was further used to determine whether the tissue margin following clinical ovarian cancer surgery contained tumor.

**Conclusion:** In combination of ratiometric fluorescence probes with imaging instrument, a point-of-care imaging method was developed and may be used for surgical navigation and rapid diagnosis of tumor tissue during clinical tumor resection.

## Introduction

Most women diagnosed with epithelial ovarian cancer (>75%) have peritoneal metastasis which caused low survival rate 1, 2. The treatment for most ovarian cancers is primarily surgical resection. The key to the success of tumor resection is accurately and rapidly locating the tumor during surgery, especially tiny metastatic tumors 3, 4. Several imaging techniques such as MRI, CT and PET/SPECT can only be used for preoperative diagnosis of cancer and a definite diagnosis during the operation process is difficult 5, 6. Histological section analysis is the gold standard in cancer diagnosis due to its high diagnostic accuracy. However, it is labor-intensive, costly and time consuming.

Fluorescent molecular imaging is considered an efficient method of localizing tumors during surgery due to its high sensitivity and biosafety 7, 8. The detection of tumor biomarkers is considered a significant method for accurate cancer detection 9-13. γ-Glutamyltranspeptidase (GGT, enzyme commission number 2.3.2.2) is a cell surface-bound enzyme 14. It has been proven that GGT is overexpressed in many different types of cancer and can serve as a biomarker for tumor tracing 15. Great efforts have been made to improve the performance of fluorescent probes for GGT detection *in vitro* and *in vivo* 16-30. However, most of these reported probes are based on the enhancement of a single band fluorescence signal, which is easily interfered by the ambient environment, including pH, probe concentration and excitation power.

The ratiometric detection technique has been demonstrated to be a more reliable method than single emission band detection. Monitoring the ratio change with two or more different emission signals can reduce the interference caused by the external environment to a great extent 31-37. To date, only several ratiometric fluorescence probes for GGT detection have been reported 22-30. Unfortunately, these probes are difficult to detecting GGT in tissue specimens, due to the strong background signal by ultraviolet excitation 22, 23, low luminescence quantum yield in aqueous solution and small emission wavelength shift caused by GGT-catalytic reaction 24-27. Therefore, construct a rapidly and highly sensitive ratiometric fluorescence probe for GGT detection in tissue sample is still a challenge.

**Scheme 1:**
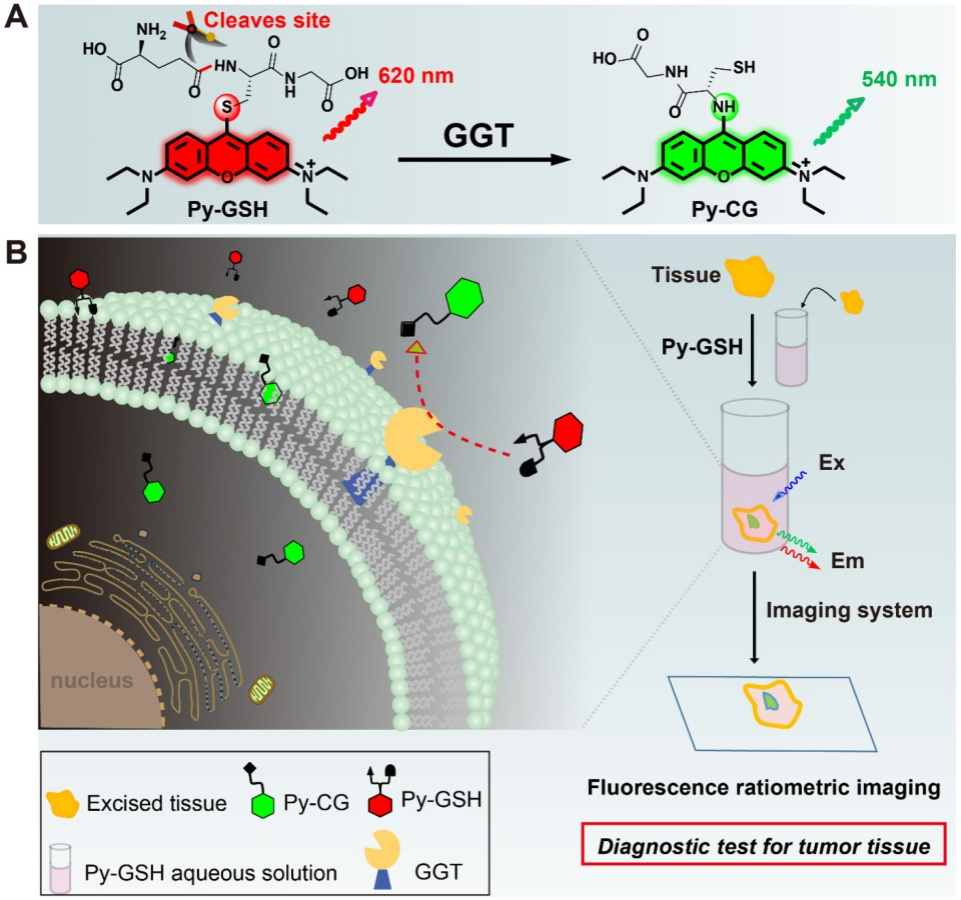
(A) Experimental design of ratiometric probe **Py-GSH** for GGT detection. Red emitting **Py-GSH** was constructed by directly linking the GSH (a GGT reactive group) to the meso-site of the fluorescent pyronin B. The presence of GGT leads to γ-glutamyl cleavage of **Py-GSH**, with the subsequent fast intramolecular rearrangement reaction of **Py-CG**, which simultaneously results in a green emission. (B) Schematic illustration of ratiometric probe in diagnostic tissue test for point-of-care tissue imaging, and the supposed process of the reaction between the probe and GGT near the suface of cancer cells.

In the present study, we developed a ratiometric GGT-responsive probe** Py-GSH** for point-of-care tissue imaging to diagnose ovarian cancer during surgery. **Py-GSH** was constructed by directly linking the GSH (a GGT reactive group) to the meso-site of the fluorescent pyronin B, and emitted strong red fluorescence. The presence of GGT leads to γ-glutamyl cleavage of **Py-GSH**, with the subsequent fast intramolecular rearrangement reaction of **Py-CG** (cysteinylglycine linked pyronin), which simultaneously results in a bright green emission (Scheme 1A). Both the probe and its GGT-catalytic product have high fluorescence quantum yield of nearly 20% in aqueous solutions. The ratio of the response signal of green emission at 545 nm to the reference signal of red emission at 620 nm was utilized to detect GGT. We found 170-fold ratio change following the activation of **Py-GSH** by GGT. Furthermore, we applied **Py-GSH** to detect ovarian cancer abdominal metastatic tumors in mice (~1 mm in diameter). Significantly, we further expanded the practical implementation of the designed** Py-GSH** to detect ovarian tumors in human specimens using simple naked eye detection or point- of-care tissue imaging (Scheme 1B). In specimens excised from 16 patients, malignant lesions had higher ratio values than normal tissues.

## Results and Discussion

### Design and Synthesis of the GGT Probe

In the biological environment, GGT plays a key role in adjusting redox homeostasis through the enzyme catalysis reaction of GSH.^7^ Accordingly, GSH can be designed as a reactive group of the GGT substrate. On the other hand, the emission wavelength of a luminophore based on the intramolecular charge transfer (ICT) is easily affected by external environments like solvents, causing an increased error in ratiometric detection. Herein, we chose pyronin B as a fluorophore, and GSH linked pyronin B was designed as the ratiometric probe for GGT detection.

In our design, the γ-glutamyl cleavage of the red emitting **Py-GSH** was specifically catalyzed by GGT and generate the green emitting product amino- modified **Py-CG** by S_N_Ar rearrangement 38, 39. Theoretical calculations indicated that the S_N_Ar rearrangement resulted in a transfer of the transition method of the excited state, which was responsible for the change in optical properties from **Py-GSH** to amino-modified **Py-CG** (Figure S1 and S2). Importantly, the fluorescence of both **Py-GSH** and **Py-CG** were mostly attribute to π*→π which resulting the influence of solvent to their emission wavelength are slight. This property of probe will benefit in ratiometric detection.

The synthesis of **Py-GSH** is shown in Scheme S1 39. Chemical structure of **Py-GSH** was characterized by ^1^H NMR, ^13^C NMR and HRMS spectra (shown in Supplemental information).

### Optical Response of Probe to GGT

**Figure 1:**
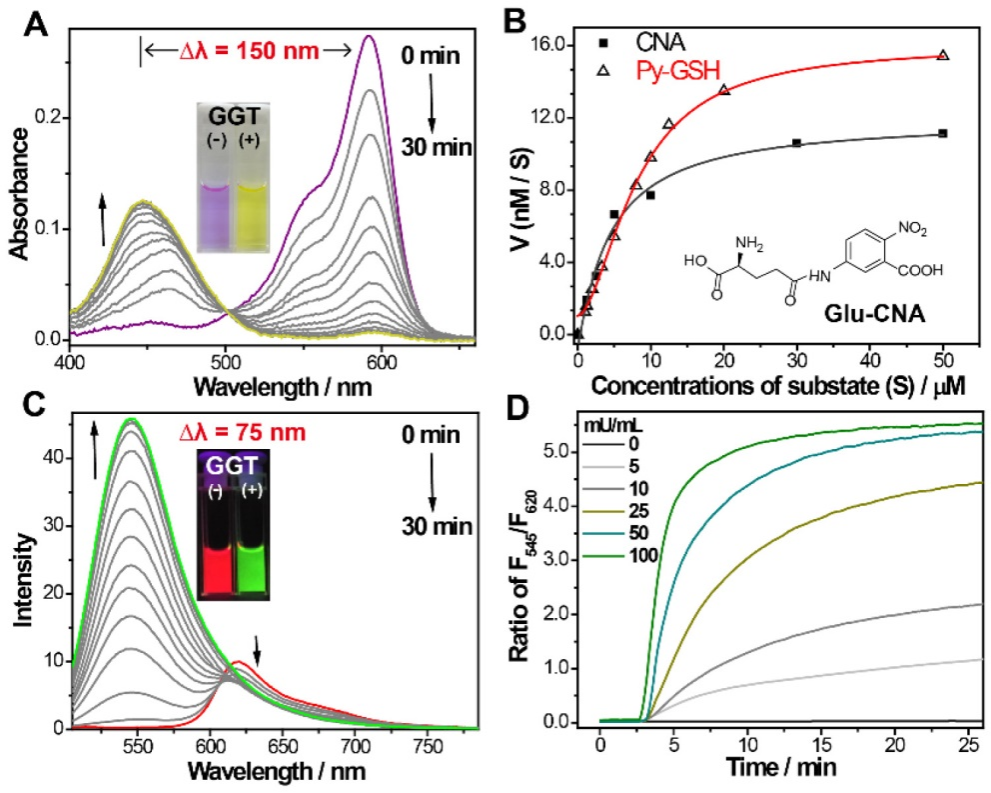
(A) Time-dependent absorption and (C) emission spectra changes of **Py-GSH** (5 µM ) in the presence of GGT (50 mU/mL) in PBS solutions (pH 7.4) at 37 ℃. Inset: photos of GGT treated **Py-GSH** solutions at 0 min and 30 min. (B) Michaelis-Menten plots of **Py-GSH** and Glu-CNA with GGT, the Glu-CNA was choosed as the reference compound. (D) Time-dependent fluorescence ratio changes of **Py-GSH** (5 μM) in the presence of different concentration of GGT. λ_ex_=488 nm.

To confirm the response of** Py-GSH** to GGT, the UV/Vis absorption and fluorescence emission spectrum of **Py-GSH** in the absence and presence of GGT were investigated in phosphate-buffered saline (PBS) solution (pH 7.4, 37 ℃). As shown in Figure 1A, the absorption bands at 593 nm gradually reduced and the absorption bands at 445 nm increased following incubation with GGT, respectively. To our knowledge, this is the largest absorption band shift of the GGT responsive probe (Table S1). In accordance with the change in absorption, the color of the solution changed from purple to yellow (inset in Figure 1A). The kinetic values of **Py-GSH** against GGT were further evaluated according to the Michaelis-Menten equation, and the K_m_ and V_max_ were calculated to be 22.6 µM and 29.7 nM/s, respectively (Figure 1B and Figure S3), which suggested high catalytic efficiency of GGT for **Py-GSH** and was very close to the GGT detection probe Glu-CNA used in the clinic. Fluorescence intensity of the emission band at 545 nm significantly increased and the emission at 620 nm weakly decreased (Figure 1C), which caused a marked ratiometric change by collecting the integration of emission intensity at 510-560 nm to 620-690 nm (170-fold ratio change) in 15 min. The large difference between these two emission wavelengths (up to 75 nm) allowed accurate measurement of the two emission intensities to yield the ratiometric value. In accordance with the emission spectrum, a clear fluorescence color change from red to green was observed with the naked eye (inset in Figure 1C).The photophysical properties of **Py-GSH** and **Py-CG** were measured in PBS (Figure S4). In addition, a faster and larger ratiometric fluorescence change in the probe was carried out by incubating in higher concentrations of GGT (Figure 1D). This indicated that the probe **Py-GSH** was capable of quantitative detecting GGT in aqueous solution. However, such fluorescence enhancement of **Py- GSH** was not induced by acivicin- treated GGT (Figure S5) due to the inhibiting effect of acivicin on GGT. These results suggest that the ratiometric fluorescence response of **Py-GSH** was activated by GGT.

**Figure 2:**
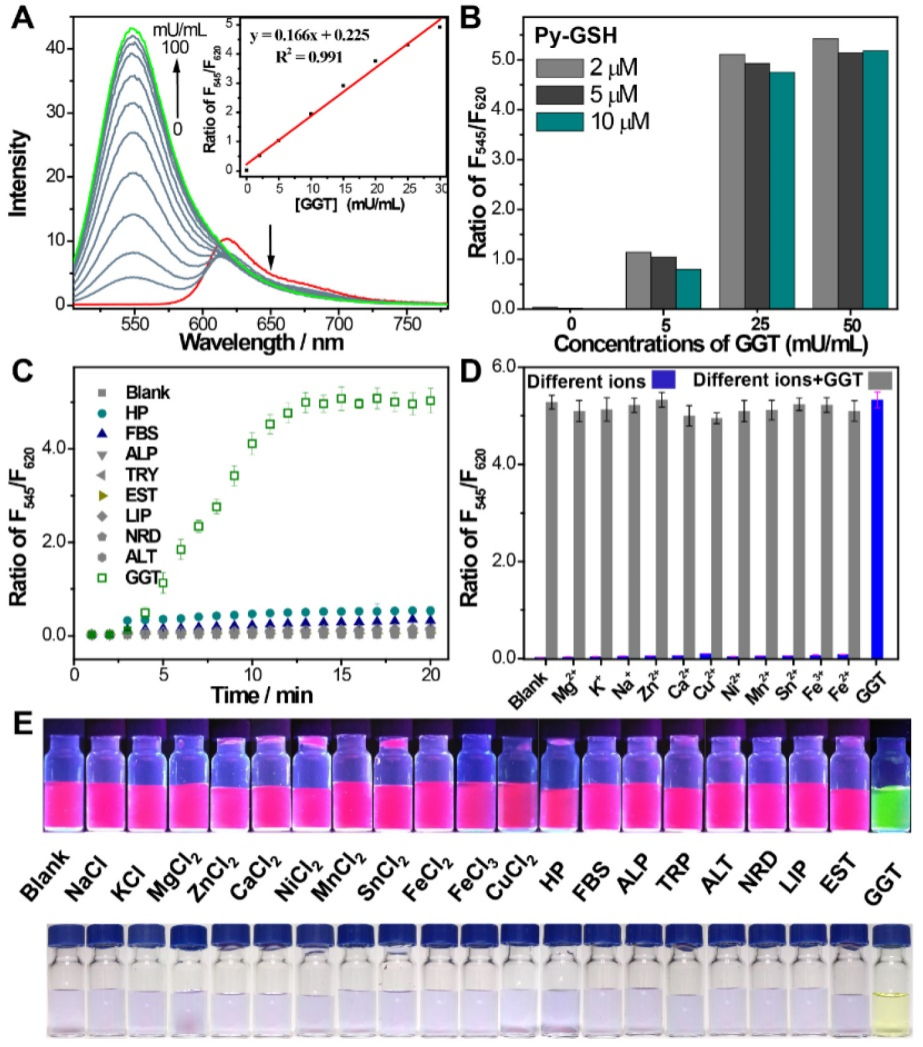
(A) Emission spectra of** Py-GSH** (5 μM) in the presence of different amount of GGT in PBS solutions (pH 7.4) at 37 ℃. Inset: corresponding plots of the fluorescence intensity ratio from 545 nm to 620 nm (F_545_/F_620_) as a function of GGT concentration. (B) F_545_/F_620_ of 2, 5, 10 μM **Py-GSH** after treated with 0, 5, 25, 50 mU/mL GGT for 30 min, respectively. (C) time dependent fluorescence ratio of F_545_/F_620_ in the presence of fetal 10% bovine serum (FBS), 5% human plasma (HP), 0.5 U/mL alkaline phosphatase (ALP), 0.2 U/mL trypsase (TRY), 0.5 U/mL esterase (EST), 0.5 U/mL lipase (LIP), 0.1 U/mL reductase (NRD), 0.2 U/mL alanine transaminase (ALT) and 0.05 U/mL γ-glutamyltranspeptidase (GGT). (D) Ratio of F_545_/F_620_ in the presence of different biological ions (NaCl-10 mM, KCl-10 mM, MgCl_2_-2.5 mM, CaCl_2_-2.5 mM, ZnCl_2_-1 mM, NiCl_2_-0.2 mM, MnCl_2_-0.1 mM, SnCl_2_-0.1 mM, FeCl_2_-0.1 mM, FeCl_3_-0.1 mM, CuCl_2_-0.1 mM) and 0.05 U/mL GGT. λ_ex_=488 nm. (e) Images of the fluorescence and color change of **Py-GSH** (5 μM) to various analytes in PBS, λ_Ex_=365 nm. (n = 3)

The response of **Py-GSH** to various concentrations of GGT was monitored by fluorescence spectra (Figure 2A and Figure S6). Enhancement of the F_545_/F_620_ value (the fluorescence intensity ratio of emission at 545 nm to emission at 620 nm) was directly proportional to the GGT concentration in the range of 1-30 mU/mL. The limit of detection of Py-GSH was measured to be 10 mU/L. With increasing concentration of **Py-GSH** from 2 μM to 10 μM, the F_545_/F_620_ values at the same GGT concentration were stable, but the fluorescence intensity at 545 nm and 620 nm at the same GGT concentration progressively increased (Figure 2B). This demonstrated that the ratiometric probe reduced the interference caused by the probe concentration. The ratiometric detection of GGT under different excitation power was performed as shown in Figure S7. At an equal concentration of **Py-GSH**, the emission intensity at 545 nm and 620 nm both depended on excitation power and GGT concentration, but the F_545_/‍F_620_ value only depended on GGT concentration. This indicated that the variation in excitation source had no influence on the ratiometric detection of GGT.

The specificity of the GGT activated ratiometric response of **Py-GSH** was verified (Figure 2C-E and S8). The fluorescence spectra of **Py-GSH** solutions were measured following the addition of a variety of enzymes and biological analytes. The color and spectrum of the solutions showed no obvious changes, except the one with added GGT. Moreover, the presence of different types of ions did not interfere with the response of **Py-GSH** to GGT (Figure 2D). Both **Py-GSH** and **Py-CG** displayed a negligible change in their fluorescence ratio of F_545_/F_620_ when the pH changed from 4.1 to 9.0, respectively (Figure S9). Therefore, the fluorescence signals of **Py-GSH** and **Py-CG** were stable in various physiological environments.

**Scheme 2:**
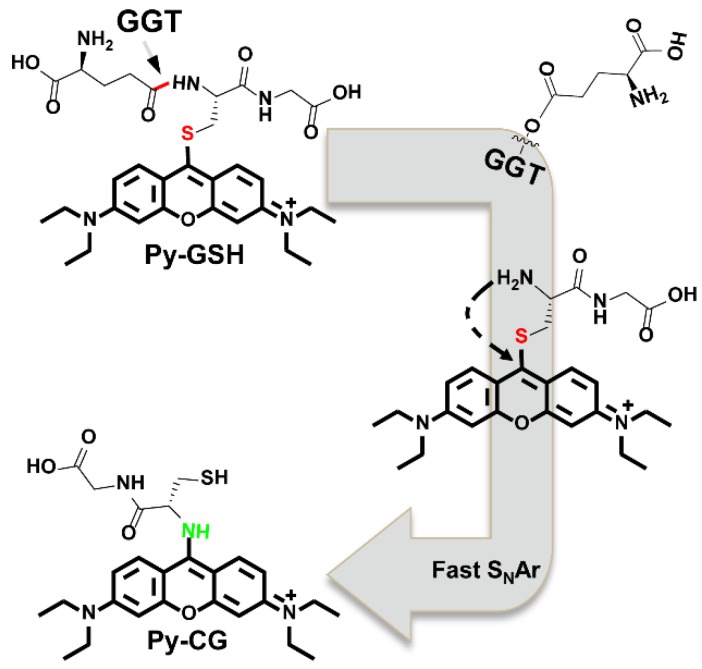
Proposed responsive mechanism of **Py-GSH** to GGT.

The response mechanism of **Py-GSH** to GGT was investigated by HPLC-MS, absorption and fluorescence spectra. The absorption and fluorescence spectra of cysteinylglycine (Cys-Gly) incubated compound **S3** solutions and GGT incubated **Py-GSH** solutions were almost the same (Figure S10), which confirmed the transfer from thiol- to amino- modified **Py-CG**. Moreover, the intramolecular substitution was also demonstrated by HPLC-MS analysis (Figure S11). As shown in Figure S11B and 11E, by mixing the solution of compound S3 and Cys-Gly, a compound in the mixture which had a retention time of 2.7 min was monitored with a mass peak at 499.23, which indicated the generation of **Py-CG**. The mass peak at 499.23 was also detected in the mixture of GGT and **Py-GSH**, and its corresponding retention time was 2.7 min (Figure S11C and 11F). It was confirmed that mixing GGT and **Py-GSH** generated **Py-CG** as the retention time of** Py-GSH** was measured to be 2.2 min and the mass peak of** Py-GSH** was measured to be 628.28 (Figure S11A, D). According to these results, we proved that GGT induced the rearrangement response mechanism shown in Scheme 2.

### Ratiometric Imaging of Cellular GGT

Prior to cellular imaging, the cytotoxicity of **Py-GSH** was evaluated. Low cytotoxicity of **Py-GSH** was observed in human ovarian cancer cells (SKOV3, CAOV3) and normal human ovarian surface epithelial cells (HOSEpiC) by 3-‍(4,5-dimethylthiazol-2-yl)-2,5-diphenyltetrazolium bromide (MTT) assay (Figure S12).

**Figure 3:**
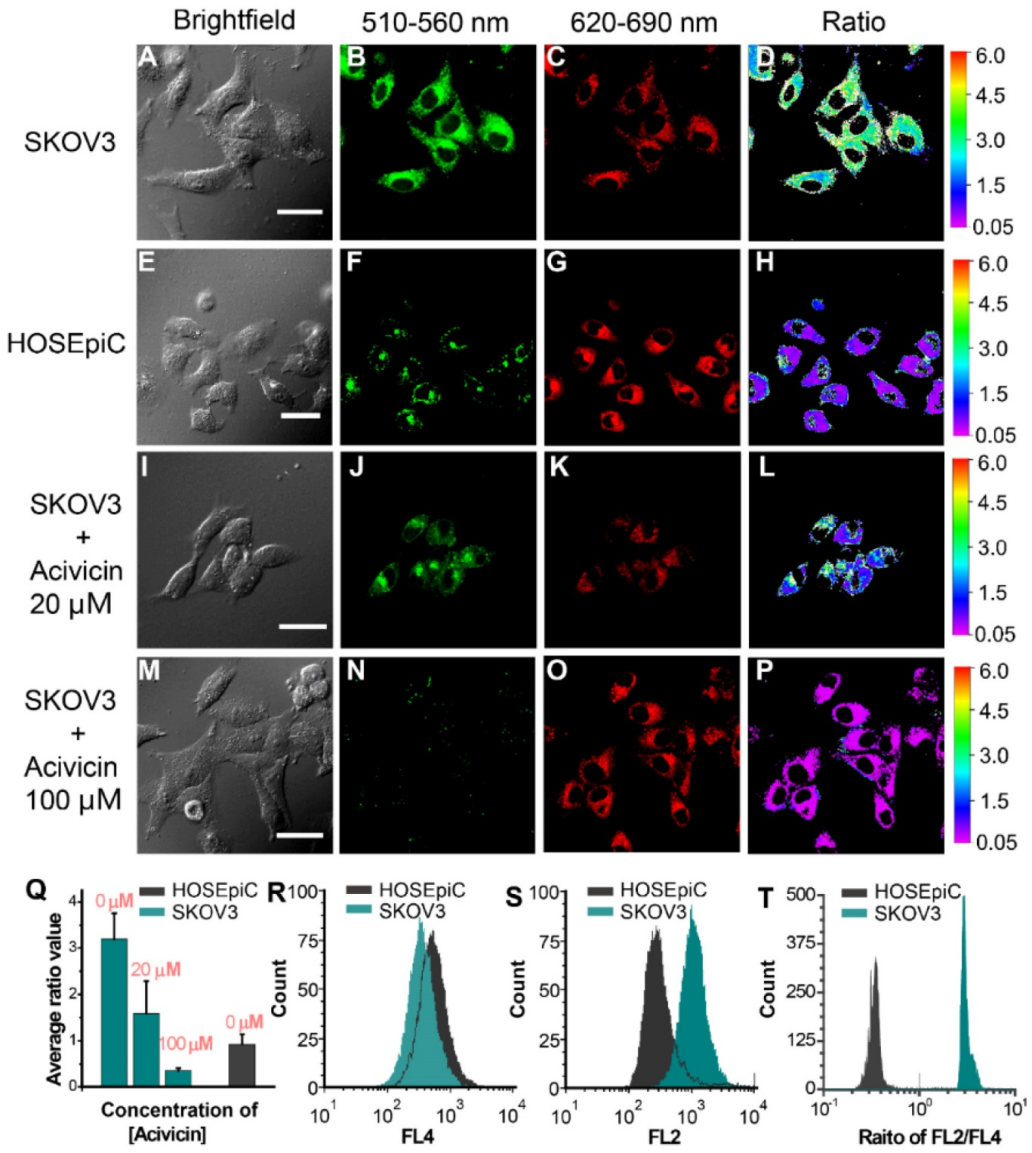
Fluorescence images of SKOV3 (A-D), HOSEpiC cells (E-H), acivicin pretreated SKOV3 (I-P) upon incubated with **Py-GSH** (5 μM) for 30 min. The emission signal of probe were collected at 510-560 nm (green channel) and 620-690 nm (red channel), respectively. The ratio image generated from green to red channel. SKOV3 were pretreated with acivicin (20 μM, 100 μM) for 30 min then incubated with **Py-GSH** (5 μM). Scale bar, 30 μm. (Q) Quantification of average ratio value in images of SKOV3, HOSEpiC and acivicin treated SKOV3. (R-T) Flow cytometric analysis of SKOV3 and HOSEpiC cells after incubated with **Py-GSH** (5 μM ) for 30 min. λ_ex_=488 nm, FL2: 560±15 nm, FL4: 675±15 nm.

The GGT level in cancer cells was investigated by laser-scanning confocal fluorescence microscopy (Figure 3A-P and Figure S13A-L). According to the ratiometric response of **Py-GSH** to GGT, we detected cellular GGT by collecting the fluorescent signal with two emission channels (green channel: 510-560 nm, red channel: 620-690 nm). The cells were incubated with **Py-GSH** 30 min before fluorescence imaging. Compared with the strong emission signal of the ovarian cancer cells in the green channel (Figure 3B and Figure S13B), emission in the red channel (Figure 3C and Figure S13C) was relatively weak. The opposite result was obtained in the control group (HOSEpiC cells), with a weak signal in the green channel (Figure 3F) and a relatively strong signal in the red channel (Figure 3G). In addition, when the cancer cells were treated with the GGT inhibitor acivicin, the green channel signal decreased with increasing acivicin concentration (Figure 3J, 3N and Figure S13F, S13J), but there was no obvious change in the red channel signal (Figure 3K, 3O and Figure S13G, S13K). These results suggested that the presence of GGT in human ovarian cancer cells (SKOV3, CAOV3) enhanced the green channel signal. According to the ratio of emission intensity generated in the green channel to that in the red channel, an obviously different emission ratio between the ovarian cancer cells and normal ovarian epithelial cells was obtained (Figure 3Q and Figure S13M). Thus, this sensitive ratiometric probe can be used to distinguish GGT positive cancer cells and GGT negative cells in living biological systems. Colocalization experiments suggested that both **Py-GSH** and generated **Py-CG** permeated the lipid bilayer of the cell membrane and accumulated mainly in the cytoplasm (Figure S14).

We studied the anti-interference ability of this ratiometric probe in GGT detection in cells using laser-scanning confocal fluorescence microscopy. Compared with the variation in a single channel, the fluorescence ratio in the green channel to that in the red channel in SKOV3 cells was relatively stable under varied excitation power and exposure time, respectively (Figure S15 and S16). In addition, the emission ratio from the two emission channels showed no significant change in SKOV3 cells incubated with different concentrations of **Py-GSH** (Figure S17). This indicated that the ratiometric probe worked in unpredictable experiment conditions and could overcome the deviations caused by the heterogeneous enrichment of the probe in tissue or *in vivo*.

To further evaluate the reliability of this ratiometric probe for monitoring cellular GGT levels, we conducted high throughput fluorescence flow cytometry assays of SKOV3, CAOV3 and HOSEpiC cells pretreated with **Py-GSH** (Figure 3R-T and Figure S13N-P). In the flow cytometry assay, two emission signals were collected at 560±15 nm (FL2) and 675±15 nm (FL4) that were excited by 488 nm. In the FL2 channel, the emission intensity of cancer cells (SKOV3, CAOV3) was stronger than that of normal cells (HOSEpiC). However, there is a partial overlap between the normal cells and cancer cells in Figure 3S (Figure S13N, S13O), which resulted in a potential deviation in the detection of cellular GGT. However, the cell lines were clearly distinguished by the ratio of each cell from the emission intensity of FL2 to FL4 (Figure 3T and Figure S13P). It is suggested that the ratiometric probe was able to accurately detect GGT levels in different cell lines.

### Ratiometric Imaging of Tumor GGT in Tissue

**Figure 4:**
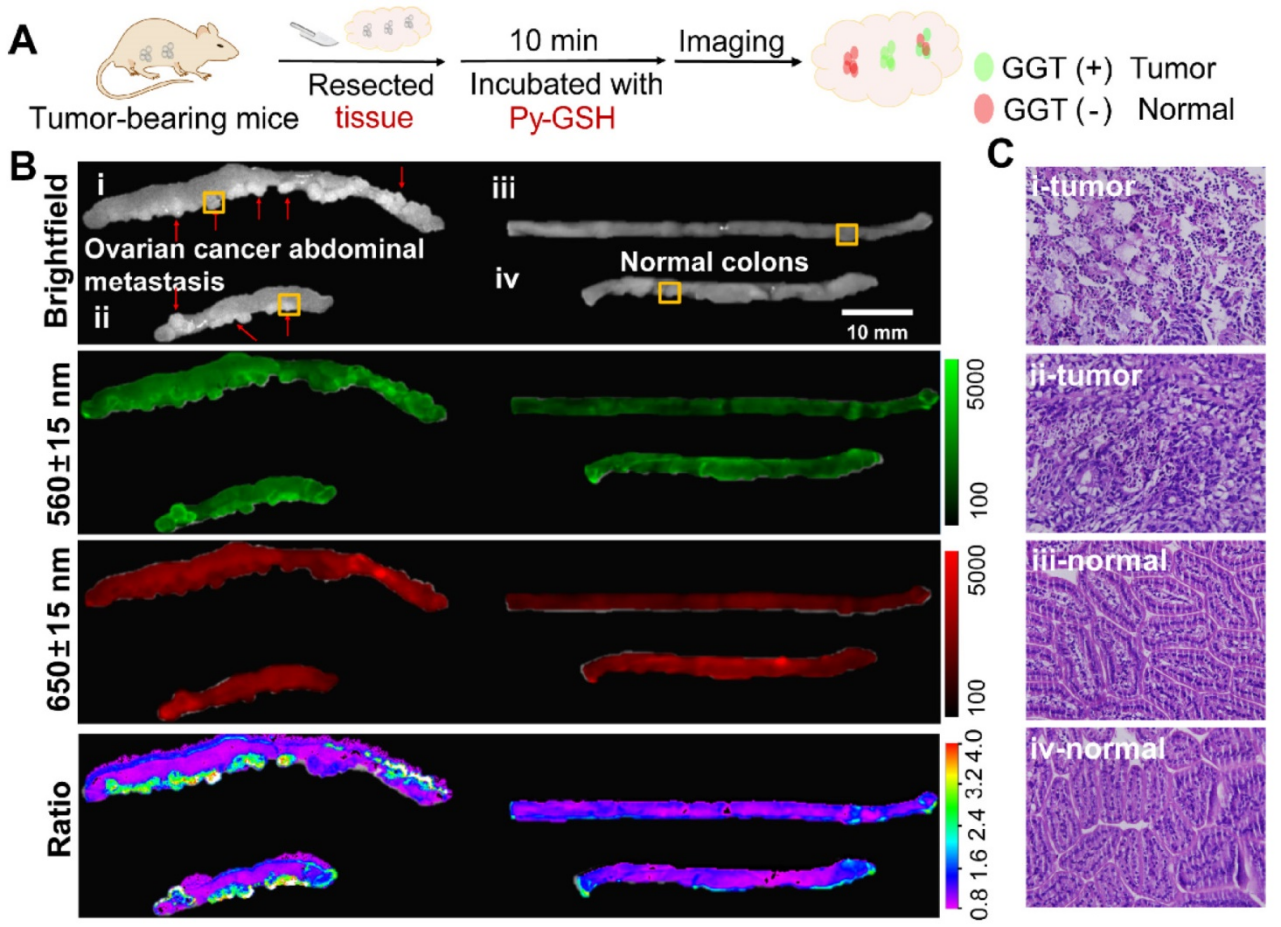
(A) Schematic illustration for detection of abdominal metastasis tumor. (B) Fluorescence images of the colon tissues of abdominal metastasis tumor-bearing mice and normal mice after stain with 10 μM **Py-GSH** saline for 10 min. In fluorescence imaging, the emission channel at 560±15 nm (Green channel) and 650±15 nm (Red channel) were collected. In ratiometric imaging, the ratio of emission intensity at 560±15 nm to that at 650±15 nm was chosen as the detected signal. λ_ex_=488 nm. (C) H&E staining specimen of yellow panel labeled tumor mice and normal mice tissue as shown in (B).

Our previous experiment demonstrated that **Py-GSH** displayed a distinctly different fluorescence change in ovarian cancer cells (SKOV3, CAOV3) and normal ovarian epithelial cells (HOSEpiC). The capability of **Py-GSH** for detecting GGT activity in solid tumor tissues and small peritoneal metastatic tumors was examined (Figure 4 and Figure S18). Firstly, we constructed a solid tumor model of ovarian cancer in mice by subcutaneous injection and constructed an intraabdominal metastasis model in mice by intraperitoneal injection (Figure 4A). We then simulated the process of clinical surgery to remove the tumor tissue. However, different to the clinical time-consuming frozen section analysis to determine the cutting edge, we removed the entire piece of tissue directly as the cut edge detection sample using the ratio of fluorescence imaging to determine whether there was a metastatic lesion.

As shown in Figure 4B and Figure S18, the tissue containing tumors and other normal organs were collected immediately after sacrifice, and then incubated with 10 μM **Py-GSH** at 37 ℃, respectively. Ten minutes later, fluorescence imaging of the tissues was performed using a modified imaging system. According to the different signal intensity of the green channel (560±15 nm), we easily found the solid tumor tissues, but it was difficult to differentiate tiny metastatic tumor tissue from normal intestinal tissue. In addition, the fluorescence intensity of the green channel in lung tissue was stronger than that in the other four organs. Therefore, false positive results showing that GGT activity of lung tissue was higher than that of the other four organs were obtained (green channel in Figure S18 and S20). This was mainly caused by different concentrations of fluorescent probes and the variation in microenvironment in different tissues. In the red channel at 650±15 nm, we found that the fluorescent signal of lung tissue was strongest (red channel in Figure S18 and S20), which suggested that lung tissue may accumulate more probes or the fluorescent probe in the lung tissue is brighter. Compared with the other organs, the kidney exhibited a higher ratio value and the lung displayed a lower ratio value, which indicated a higher level of GGT in the kidney and lower GGT activity in the lung. These results are in accordance with previous reports.^22^ According to the ratio images, tumor lesions were clearly distinguished as the tumor tissue exhibited a higher ratio value than normal organs and tissues. Small metastases in the colon were clearly identified (Figure 4B) and H&E staining results further demonstrated that this region contained tumor tissue (Figure 4C, Figure S19 and S21). Compared with the normal tissue ratio value which was less than 1, in most tumor tissue the ratio value was greater than 2.5, which is consistent with previous cellular imaging experiments (Figure 3Q). Such an obvious difference makes it easier to distinguish tumor tissue and normal tissue using ratiometric imaging. This demonstrates that **Py-GSH** has a promising application for monitoring micro-tumor lesions in clinical specimens.

### Ratiometric Imaging of Clinical Specimens

**Figure 5:**
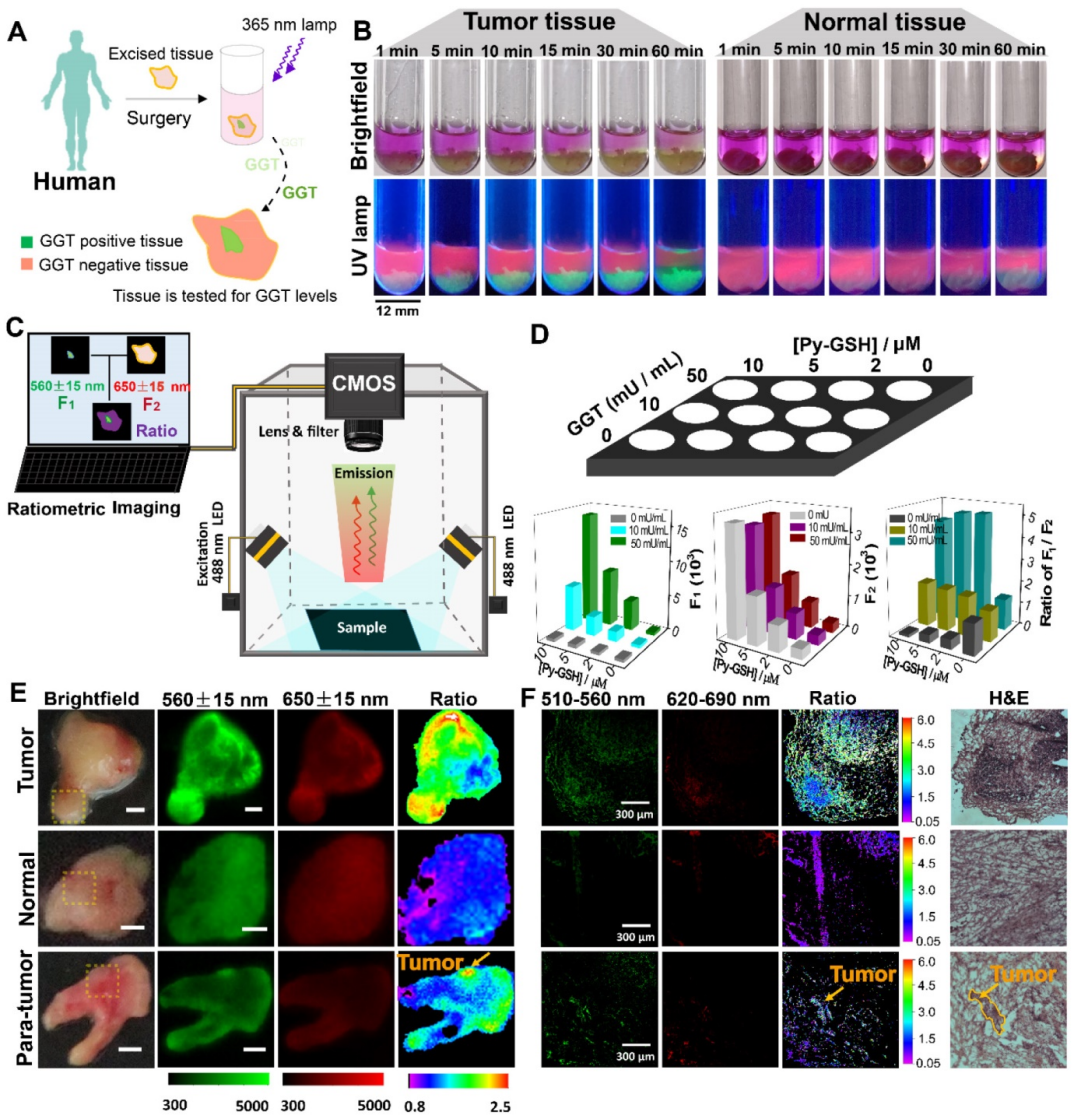
(A) Schematic illustration for GGT detection in human specimens. (B) Time-dependent photo images of different tissues of tumor and normal tissues treated with 10 μM Py-GSH saline for 60 min. Excitation source, 365 nm lamp. (C) Structure diagram of the ratiometric fluorescence imaging system in diagnostic tissue test for human specimens and *in vitro* anti-interference test. (D) Simplified diagram depicting the experimental setup of the concentration interference experiment, the average fluorescence intensity of different signal collecting channel (Green channel, F1-560±15 nm; red channel, F2-650±15 nm) and average ratio value of every well. (E) Fluorescence images of the human tissues after stain with 10 μM **Py-GSH** saline for 10 min. In fluorescence tissue imaging, the emission channel at 560±15 nm (Green channel) and 650±15 nm (Red channel) were collected. In ratiometric imaging, the ratio of emission intensity at 560±15 nm to that at 650±15 nm was chosen as the detected signal. λ_ex_=488 nm. Scale bar, 2 mm. (F) Frozen section analysis of corresponding yellow panel labeled tissues shown in (E) and fluorescence confocal images of the adjacent slices of H&E images. The emission signal of probe were collected at 510-560 nm (green channel) and 620-690 nm (red channel), respectively. The ratio image generated from green to red channel.

**Py-GSH** was used to detect tumor lesions in freshly excised human specimens by naked eye dection and ratiometric fluorescence imaging (Figure 5). As shown in Figure 5B, the excised tissues were incubated in 10 μM **Py-GSH**, under irradiation from a 365 nm UV lamp, and tumor tissue exhibited a time-dependent increase in green fluorescence. In addition, the color of the solution around the tumor tissue gradually changed from purple to light yellow. In contrast, normal tissues and the control groups showed no obvious optical changes following 30 min incubation (Figure S22). This obvious optical change in stained tumor tissue was observed by the naked eye in just 5 min. These results indicated that this ratiometric probe may have potential application in the rapid identification of cancer tissues by the naked eye.

The accuracy of this ratiometric probe with interference due to probe concentration and the effect of tissues under imaging conditions was investigated. Fluorescence imaging was performed using a modified imaging system (Figure 5C). The luminescence signals were collected at 560±15 nm (green channel) and 650±15 nm (red channel), respectively, which corresponded to the emission band of **Py-CG** and **Py-GSH**. With increasing GGT concentration and the same concentration of **Py-GSH**, the fluorescence intensity in the green channel was obviously enhanced, while the intensity in the red channel did not change (Figure 5D and Figure S23). Additionally, at the same concentration of GGT, both the fluorescence signal in the green channel and red channel showed increased intensity with increasing **Py-GSH** concentration. These changes depended on GGT concentration. Therefore, the various concentrations of **Py-GSH** interfered with the accuracy of GGT detection when a single emission band was used as the collected signal and the ratiometric probe reduced the interference caused by probe concentration. In contrast to tissue-free images, when pork tissue (1 mm) covered the well plate (Figure S24), fluorescence intensity in both the green and red channel decreased in most wells due to tissue absorption and scattering. The collected fluorescence signals from both channels showed similar variation regarding the concentration change in **Py-GSH** or GGT shown in Figure 5D. However, the ratio value in the green channel to that in the red channel was also independent of the **Py-GSH** concentration (Figure S24C). The slight difference in ratio values shown in Figure 5D and Figure S24 may have been caused by interference due to auto-fluorescence of the tissue.

Ratiometric fluorescence imaging was carried out on human specimens following incubation with **Py-GSH** for 10 min. As shown in Figure 5E, the ratiometric fluorescence images of tumor tissue showed a higher ratio value than normal tissue. According to these different ratio values, the tumor lesions were clearly identified. Compared with the H&E stained images and fluorescence confocal images, it was found that the tumor lesions corresponded to the regions with high ratio values in ratiometric imaging (Figure 5F). Based on these results, using specimens excised from 16 patients, the fluorescence ratiometric change after 10 min incubation with **Py-GSH** in various ovarian lesions and adjacent normal tissues were then measured. As shown in Figure 5E and Figure S25, malignant lesions had higher ratio values than normal tissues. These results indicated the validity of this ratiometric fluorescence-based method as a clinical tool for intraoperative tumor margin assessment.

## Conclusion

In summary, we designed a fluorescent ratiometric probe (**Py-GSH**) consisting of GSH (reactive group for GGT) and pyronin B (fluorescent reporter) for monitoring GGT activity. Compared to reported ratiometric probes, **Py-GSH** was demonstrated to be a ratiometric fluorescence probe with high sensitivity (>170-fold ratio change within 15 min) and low cytotoxicity in living cells. The ratiometric probe improved the accuracy of GGT detection by effectively eliminating interference due to the ambient environment. Thus, it was possible to clearly differentiate ovarian cancer cells from normal ovarian cells in real time cellular imaging and high throughout flow cytometry assay, and it was successfully performed in detecting the high GGT activity of solid tumor tissues and small peritoneal metastatic tumors (~1 mm in diameter). In addition, we constructed a corresponding ratiometric fluorescence imaging system for tissue imaging, and this probe was used to determine whether the tissue margin in clinical ovarian cancer surgery contained tumor. In specimens excised from 16 patients, malignant lesions had higher ratio values than normal tissues. Therefore, this ratiometric probe can be used to aid the detection of tiny cancerous nodules for tumor resection. In addition, chemical substitution of γ-glutamyl moiety by other acyl group may potential applied for ratiometric detecting other peptidase that overexpressed in various disease Thus, we expected that such a simple molecular design principle is capable of developing a variety of highly sensitive and anti-interfere ratiometric fluorescence probes in detection of target enzymes, then constructing a reliable diagnostics for related diseases.

## Experimental Section

### Instruments and Methods

The ^1^H and ^13^C NMR spectra were measured at room temperature by using a Bruker Ultra Shield Plus 400 MHz NMR instrument. Matrix assisted laser desorption ionization-time of flight mass spectra (MALDI-TOF/TOF) were measured on an AB SCIEX 5800 system and high resolution mass spectrometry (HRMS) were conducted on Bruker McriOTOF11. UV-Vis absorption spectra were recorded on Lambda 750 (PerkinElmer, America). Photoluminescent spectra were measured using a QM40 (PTI, America) system with a xenon lamp as the excitation source. Emission lifetime studies were performed with an Edinburgh FL920 photocounting system with a semiconductor laser as the excitation source.

### Materials

All chemicals were purchased from commercial sources and used without further purification. All solvents were purified before use. Glutamyltranspeptidase from equine kidney and GGT inhibitor acivicin were purchased from Sigma-Aldrich Co. Ltd. Pyronine B were purchased from J&K.

### Synthesis and Characterization of Py-GSH

The mixture of GSH (35 mg, 1.1 mmol) in H_2_O/CH_3_OH (2:1, 10 mL) was stirred at room temperature for 10 min then added dropwise with 2 mL S3 CH_3_OH solution (20 mg / mL). The mixture was stirred 12 h at room temperature, then concentrated to remove the solvents. The residue was purified by HPLC with CH_3_CN/H_2_O (2/1, v/v) to afford the pure product Py-GSH. Yield: 10 mg (23%). ^1^H NMR (400 MHz, D_2_O, δ) 7.77 (d, J = 9.6 Hz, 2H, Ar-CH), 6.87 (dd, J = 9.6 Hz, J = 2 Hz, 2H, Ar-CH), 6.46 (d, J = 2 Hz, 2H,Ar-CH), 4.31 (d, J = 8 Hz, 1 H,CH), 3.6-3.2 (m, 12 H, 6×CH_2_), 2.3-2.05 (m, 3H,CH2 and CH ), 1.9-1.8 (m, 2H, CH_2_), 1.21 (t, J = 7.2 Hz, 12H, 4×CH_3_). ^13^C NMR (125 MHz, D_2_O, δ) 12.1, 25.9, 31.4, 45.9, 53.8, 95.7, 114.4, 115.3, 130.6, 153.7, 155.4, 155.8, 169.0, 170.5, 172.4, 174.2. HR-MS (ESI) *m/z*: calcd for C_31_H_42_N_5_O_7_S 628.2799, found 628.2781.

### Confocal Fluorescence Imaging

Confocal fluorescence imaging was carried out on an Olympus FV1000 laser scanning confocal microscope equipped with a 60 immersion objective len. A semiconductor laser at 488 nm was served as excitation of the cells incubated with Py-GSH. The emission was collected at 510-560 nm and 620-690 nm for the cells incubated with Py-GSH. Py-GSH probe was added to DMEM to yield 5 μM solution. The cells were incubated with the Py-GSH for 0.5 h at 37 °C.

### Flow Cytomctric Assay

The SKOV3, CAOV3 and HOSEpiC cells were seeded in the six-well plates at a density of ~1 × 10^5^ for 24 h, 37 °C. Then the cells were washed once with PBS. DMEM (2 mL) with 5 μM Py-GSH was added for 30 min further incubation. A 488 nm argon ion laser was used for excitation. Signals from cells were collected with a 560±15 and 675±15 nm band-pass filter. Cells were analyzed in a FACScan cytometer (Becton Dickinson), and all data were analyzed with CellQuest software (Becton Dickinson). The mean fluorescence intensity was determined in triplicate.

### Tumor Xenografts

Animal experiments were conducted according to the guidelines of the Institutional Animal Care and Use Committee. Tumor cells were harvested when they reached near confluence by incubation with 0.05% trypsin-EDTA. Cells were pelleted by centrifugation and resuspended in sterile PBS. For subcutaneous tumor, the SKOV3 cells (5 × 10^6^ cells/site) were implanted subcutaneously into the four-week-old female athymic nude mice. When the tumors reached 0.5 cm in diameter (three weeks after implantation), the tumor-bearing mice were subjected to imaging studies. For metastasis tumor of abdomen, the SKOV3 cells (5 × 10^6^ cells/site) were implanted intraperitoneally into the four-week-old female athymic nude mice. After six weeks, the tumor- bearing mice were subjected to imaging studies.

### Fluorescence Imaging in Tissue and *in vitro*

Fluorescence imaging was performed using modified imaging system. The LED lamp (2 W) was used as the excited source, collocating with a bandpass filter (490±5 nm). The luminescence signals were collected at 560±15 nm and 650±15 nm. Images of luminescent signals were analyzed with Bruker MISE Software. The tissues were harvest from mice immediately incubated with the Py-GSH (10 μM, PBS) for 10 min at 37 °C before imaging.

### Clinical Samples

A group of 16 patients (positive group-12 patients with high grade serous adenocarcinoma of ovarian cancer, nagetive group-2 patients with ovarian endometriosis and 2 patient with ovarian lipoma) underwent surgical treatment of ovarian disease in the Obstetrics and Gynecology Hospital of Fudan University between september 2018 and october 2018. All 16 patients underwent frozen section analysis. Tumor specimens were collected intraoperatively and ex vivo fluorescence images were collected with an *in vivo* imaging system. After imaging, specimens of cryopreserved in liquid nitrogen, for use in the preparation of frozen section. All clinical data were obtained from medical and imaging records. Informed consent was obtained from all patients, and this study was approved by the ethics committees of Fudan University.

### Histological Assessment

All animal procedures were in agreement with the guidelines of the Institutional Animal Care and Use Committee, Department of Pharmacy, Fudan University. For hematoxylin and eosin (H&E) stained tissues, tumors were harvest from mice immediately. Excised specimens were immediately fixed with 10% formaldehyde for at least 24 hours. Paraffin- embedded sections were stained with hematoxylin and eosin for histopathological evaluation. The sections were observed under an optical microscope.

## Supplementary Material

Supplementary figures and tables.

## Abbreviations

GSH: glutathione
GGT: γ glutamyltranspeptidase
MTT: 3-‍(4,5-dimethylthiazol-2-yl)-2,5-diphenyltetrazolium bromide
