# Supplementary material for: Point-of-care Ratiometric Fluorescence Imaging of Tissue for the Diagnosis of Ovarian Cancer
Source: Theranostics. 2019 Jun 24;9(16):4597–607. doi: 10.7150/thno.35322 (PMC6643432; doi:10.7150/thno.35322)
Supplement: Supplementary file 1 — Supplementary figures and tables. [file thnov09p4597s1.pdf]

Supporting Information

Point-of-care Ratiometric Fluorescence Imaging of Tissue for the Diagnosis of Ovarian Cancer

Xiaobo Zhou,[†] Yawei Liu,[†] Qiyu Liu,[‡] Luzhe Yan,[§] Meng Xue,[†] Wei Yuan,[†] Mei Shi,[†] Wei Feng,[†] Congjian Xu,*[‡] Fuyou Li *[†]

† Department of Chemistry & State Key Laboratory of Molecular Engineering of Polymers & Institute of Biomedicine Science, Fudan University, Shanghai 200433, China

‡ Department of Obstetrics and Gynecology of Shanghai Medical School & Shanghai Key Laboratory of Female Reproductive Endocrine Related Diseases & Obstetrics and Gynecology Hospital of Fudan University, Fudan University Shanghai 200011, China

§ The High School Affiliated to Renmin University of China, Beijing 100080, China

*Email: fyli@fudan.edu.cn; xucongjian@fudan.edu.cn

Supplemental Experimental Procedures

Theoretical calculations

Cell culture

Cytotoxicity test

Supplementary Scheme

Scheme S1: Synthetic routine of the ***Py-GSH***

Supplementary Figure. 1-25

Figure S1: Theoretical calculation of Py-GSH and Py-CG

Figure S2: Solvent effect to the photophysical properties of Py-GSH and Py-CG

Figure S3: Kinetic characteristics of Py-GSH and Glu-CNA

Figure S4: Photophysical properties of Py-GSH and GGT incubated Py-GSH

Figure S5: Inhibition of GGT-induced fluorescence changes by acivicin

Figure S6: Interference of probe concentration for ratiometric detection

Figure S7: Interference of excitation power for ratiometric detection

Figure S8: Selectivity of Py-GSH for GGT detection.

Figure S9: Interference of pH for ratiometric detection

Figure S10: Demonstration of the response mechanism of Py-GSH to GGT by spectroscopic studies

Figure S11: Demonstration of the response mechanism of Py-GSH to GGT by HPLC-MS studies

Figure S12: Cytotoxicity of Py-GSH

Figure S13: Detection of GGT levels in CAOV3

Figure S14: Distribution of probe in cells

Figure S15: Interference of excitation power on the detection of cellular GGT

Figure S16：Interference of exposure time on the detection of cellular GGT

Figure S17：Interference of probe concentration on the ratiometric detection of cellular GGT

Figure S18：Py-GSH for ratiometric detection GGT in organs and solid tumor from tumor mice

Figure S19：H&E staining specimen of tissues shown in Figure S19

Figure S20：Py-GSH for ratiometric detection GGT in organ from normal mice.

Figure S21：H&E staining specimen of tissues shown in Figure S21

Figure S22：Time-dependent photo images of control groups

Figure S23: Interference of probe concentration on the ratiometric detection of GGT in vitro

Figure S24：Interference of tissue cover on the ratiometric detection of GGT in vitro

Figure S25：Ratiometric fluorescence images of clinical specimens

Supplementary Table 1

Chemical structure of reported GGT-activatable fluorescence probes and their photophysical propertie

NMR spectra of compound

^{1}H NMR and ^{13}C NMR of compound S2

^{1}H NMR and ^{13}C NMR of compound S3

^{1}H NMR and ^{13}C NMR of Py-GSH

Supplemental Experimental Procedures

Theoretical calculations. The structure optimization of compound was performed with the Gaussian 03 package using B3LYP density functional theory (DFT). The 6-31G(d) basis set was used to treat all atoms. The contours of the molecular orbitals were plotted. On the basis of ground- and excited-state optimization, the time-dependent density functional theory (TDDFT) approach was applied to predict their absorption and emission properties. The solvent effect (CH_2Cl_2) was simulated using the polarizable continuum model (PCM) in which the solvent cavity is regarded as a union of interlocking atomic spheres.

Cell culture. The cell lines SKOV3, CAOV3 and HOSEpiC were provided by the Institute of Biochemistry and Cell Biology, SIBS, CAS (China). The cells were grown in DMEM (modified Eagle's medium) supplemented with 10% FBS (fetal bovine serum) at 37 ºC and 5% CO_2. All cells were planted on 14 mm glass coverslips and keep to adhere for 24 h

Cytotoxicity test. The in vitro cytotoxicity was measured using a standard methyl thiazolyl tetrazolium (MTT, Sigma Aldrich) assay in SKOV3, CAOV3 and HOSEpiC cell lines. Briefly, cells growing in log phase were seeded into 96-well cell culture plate at 1×10^4/well. Py-GSH was added to the wells of the treatment group at concentrations of 2, 5, 10, 25, 50 μM/mL. For the negative control group, 1 μL/well solvent was diluted in DMEM with the final concentration of 1 %. The cells were incubated for 24 h at 37 °C under 5 % CO_2. The combined MTT/PBS solution was added to each well of the 96-well assay plate and incubated for an additional 4 h. After removal of the culture solution, 200 μL DMSO was added to each well, shaking for 10 min at shaking table. An enzyme-linked immunosorbent assay (ELISA) reader was used to measure the OD570 (absorbance value) of each well referenced at 490 nm. The following formula was used to calculate the viability of cell growth:

Viability (%) = (mean of absorbance value of treatment group / mean of absorbance value of control) × 100

Synthetic routine of the Py-GSH[1,2]

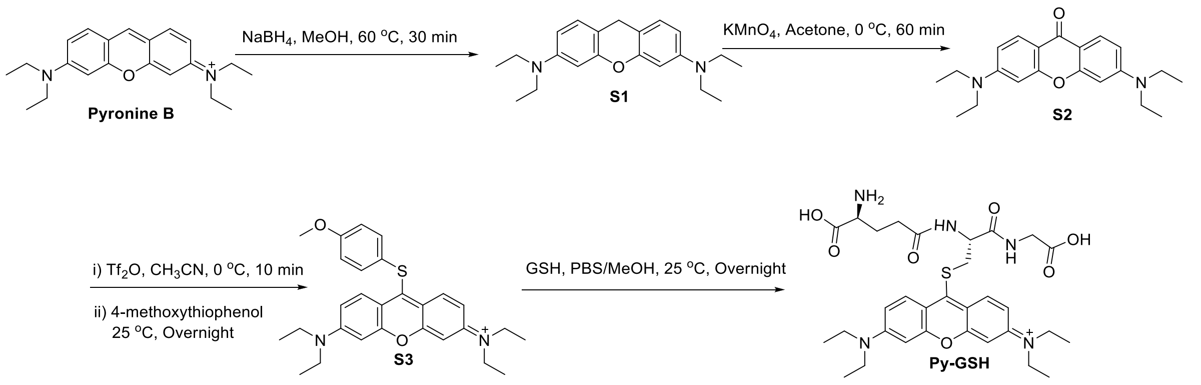

Scheme S1. Synthetic routine of the **Py-GSH**.

*Compound S1***.** 2.00 g (3.8 mmol) Pyronin B was suspended in methanol (300 mL). The solution was heated to 60 °C for 30 min. To the solution was slowly added 6 x 300 mg (6 x 6 mmol, 6 x 1 equiv.) sodium borohydride over the course of 15 minutes. Following stirred the solutions for another 30 minutes, cooled to room temperature, then evaporated all of the solvent. The violet residue was taken up in 100 mL water and 100 mL dichloromethane and the organic layer collected. The aqueous layer was further extracted with 2 x 150 mL DCM, dried over Na_2SO_4, and concentrated to yield (0.93 g, 1.9 mmol, 47%) S1 as a magenta solid and used without further purification to the next step.

Compound S2. 0.5 g (1.05 mmol) **S1** was dissolved in 25 mL acetone. The solution purged with Ar, and cooled to 0 °C. To the solution was added 3 x 100 mg potassium permanganate over 30 minutes. After 15 minutes, TLC analysis showed full consumption of starting material. The solution was then filtered over a pad of Celite, the pad washed with 100 mL acetone, and concentrated under vacuum. The resulting solid as purified by silica gel chromatography with dichloromethane to 30:1 CH_2Cl_2/MeOH to yield S2 (0.29 g, 0.568 mmol, 57%) as a red-orange solid ^{1}H NMR (400 MHz, $CDCl_3$, δ): 8.12 (d, J = 8.8 Hz, 2H), 6.67 (dd, J1 = 2.6 Hz, J2= 9.0 Hz, 2H), 6.69 (s, 2H), 3.47 (q, J = 7.2 Hz, 8H), 1.26 (t, J = 7.2 Hz, 12H) ; ^{13}C NMR (125 MHz, $CDCl_3$) δ 177.0, 161.402, 154.9, 130.7, 114.5, 111.5, 99.2, 47.6, 15.4; MS (MALDI-TOF) *m/z*: calcd for $C_{21}H_{26}N_2O_2$ 338.1994, Found 338.3152.

Compound S3. The mixture of S2 (0.2 g, 0.6 mmol) in CH_2Cl_2 (10 mL) was stirred at 0 ℃ under N_2 for 10 min then Tf_2O (200 μL, 1.2 mmol) was added dropwise over 1 min. The reaction mixture was stirred for 10 min then 4-methoxythiophenol (0.84 g, 6 mmol) was added. The mixture was stirred overnight at room temperatrue. The solvents were removed under reduced pressure and the residue was purified by flash chromatography (CH_2Cl_2/MeOH = 20/1) to afford the pure product 1 (80 mg, 44.3% yield) ^{1}H NMR(400 MHz, CD_3CN, δ) 8.10 (dd, J_1 = 3.0 Hz, J_2= 9.6 Hz, 2H), 7.40(d, J = 8.4 Hz, 2H), 6.98 (d, J = 9.6 Hz, 2H), 6.92 (d, J = 8.4 Hz, 2H), 6.72 (s, 2H), 3.79 (s, 3H), 3.63(q, J = 7.2 Hz, 8H), 1.27(t, J = 7.2 Hz, 12H) ; ^{13}C NMR (125 MHz, CD_3CN,δ) 163.2, 159.9, 158.7, 158.4, 135.8, 133.8, 127.0, 118.5, 117.9, 117.2, 98.7, 58.1, 48.6, 14.8; MS (MALDI-TOF) *m/z*: calcd for $C_{28}H_{33}N_2O_2S$ 461.2257, Found 461.4061

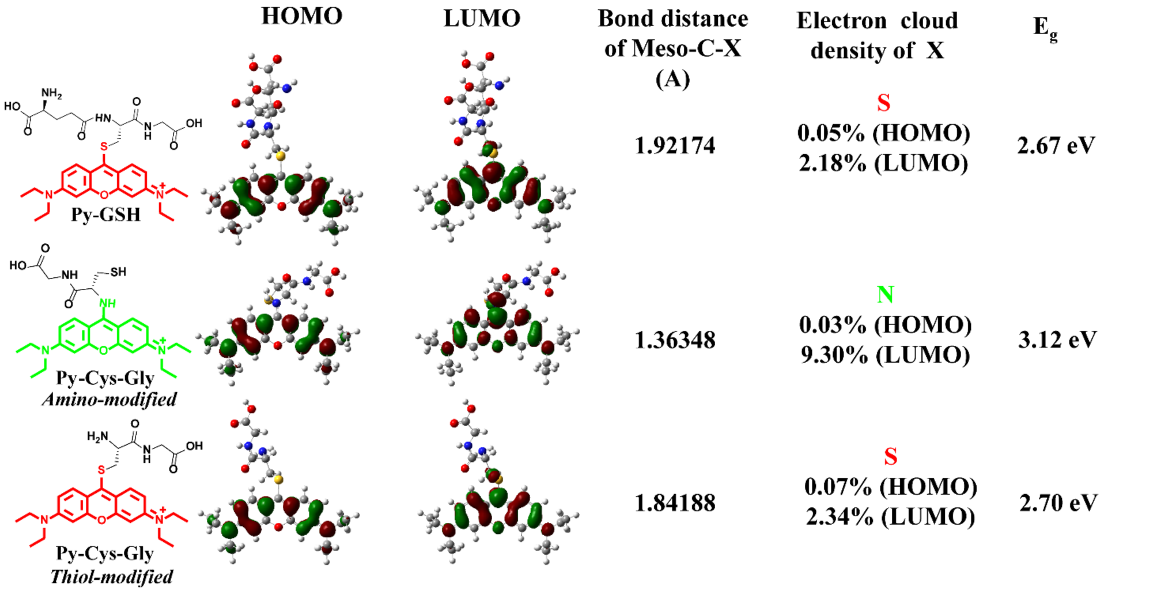

Figure S1. Calculated obital distribution, bond distance of Meso-C to the linked heteroatom (S or N), electron cloud density of meso-C linked heteroatom (S or N) and the energy gap from HOMO to LUMO of Py-GSH, amino-modified Py-CG and thiol-modified Py-CG, respectively.

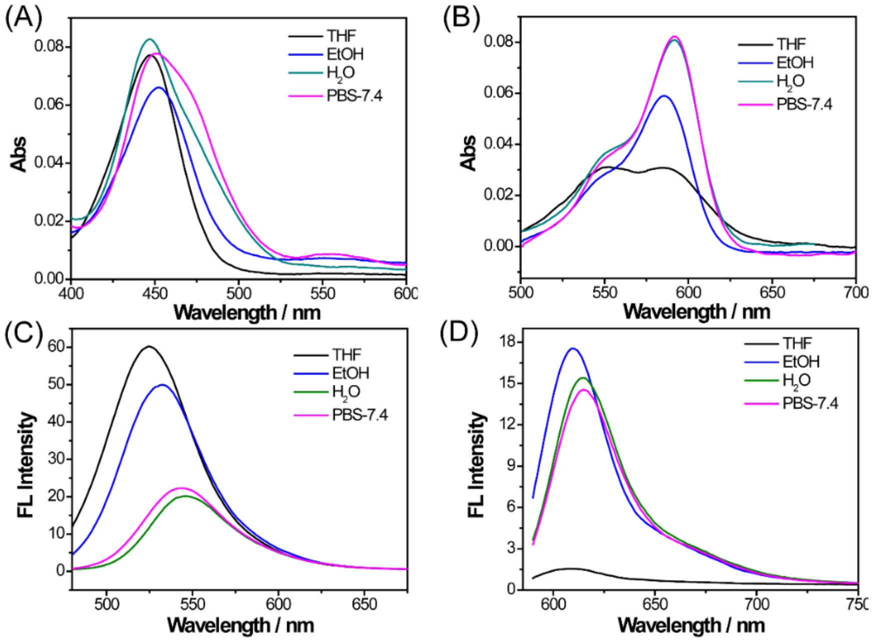

Figure S2. Absorption spectrum and emission spectrum of amino-modified Py-CG (A, C) and Py-GSH (B, D) on different solvent.

For a better understanding of the optical change from **Py-GSH** to amino-modified **Py-CG**, theoretical calculations were performed using the Gaussian 03 package at the B3LYP level [3]. As shown in Figure S1, both the highest occupied molecular orbital (HOMO) and lowest unoccupied molecular orbital (LUMO) of **Py-GSH** and thiol-modified **Py-CG** were mainly distributed over the entire conjugated backbone. Furthermore, the excited state of **Py-GSH** was clarified through time-dependent density functional theory (TDDFT) calculations, and its lowest excited state was assigned to the HOMO-LUMO transition. According to the orbital distributions, no evident charge transfer was observed. We studied the dependence of both the absorption spectra and emission property of **Py-GSH** on the solvent (Figure S2). There was no evident change in the absorption spectra and emission property in different solvents, which demonstrated that the optical property of **Py-GSH** was attributed to the transition of π-π^*. According to the same calculation method, the lowest excited state of amino-modified **Py-CG** was assigned to HOMO – LUMO, and the two orbitals were still mainly distributed over the entire conjugated backbone. However, an evident change in charge distribution on the meso-N of amino-

modified **Py-CG** from HOMO to LUMO was obtained. These findings indicated that the S_NAr substitution-rearrangement reaction resulted in a transfer of the transition method of the excited state, which was responsible for the change in optical properties from **Py-GSH** to amino-modified **Py-CG**.

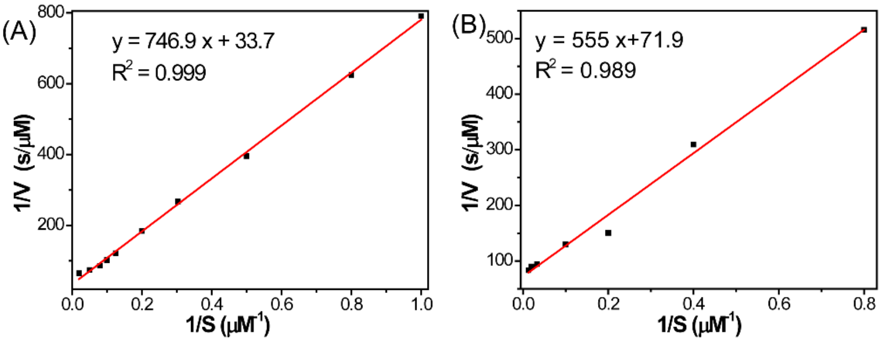

(C)

	Pyronin-GSH	Glu-CNA
K_m (μM)	22.6	7.7
V_{max} (nM/s)	29.7	13.9
K_{cat} (s^{-1})	0.453	0.212
K_{cat}/K_m ($M^{-1}S^{-1}$)	20063	27460

Figure S3. Kinetic characteristics of Py-GSH compared with Glu-CNA. Michaelis-Menten plots of 1/V as function of 1/S, (A) Py-GSH and (B) Glu-CNA. All experiments were carried out at 37 °C in phosphate buffer (pH 7.4) containing GGT (50 mU). The initial velocities were calculated from the change of absorbance of Py-GSH and Glu-CNA. (C) Chemical structure of Glu-CNA and kinetic parameters of GGT probes according to the Michaelis-Menten equation.

$$\mathbf{V} = \mathbf{V_{max}} \times [\mathbf{S}]/(\mathbf{K_m} + [\mathbf{S}]) \quad (1)$$

$$\mathbf{1/V} = (\mathbf{K_m/V_m}) \times (\mathbf{1/[S]}) + \mathbf{1/V_{max}} \quad (2)$$

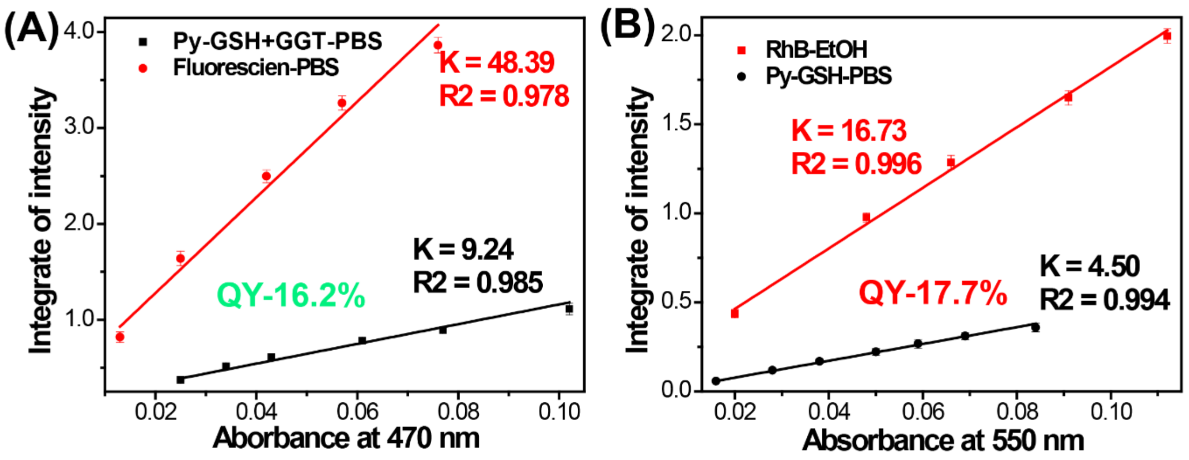

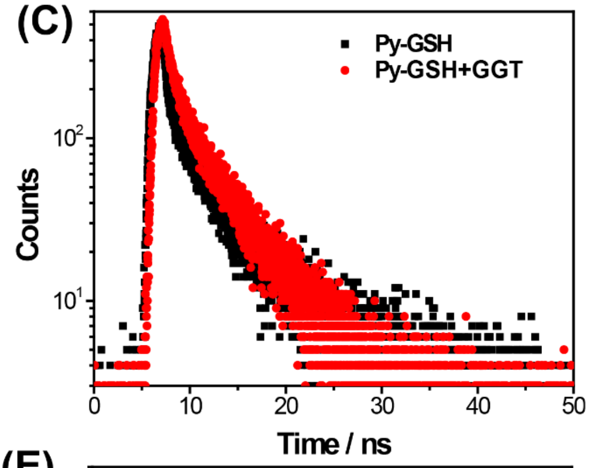

(D)

	Py-GSH	Py-GSH+GGT
λ_{max}	590 nm	455 nm
ε / $M^{-1}cm^{-1}$	53000	29000
λ_{em}	620 nm	545 nm
ϕ	16.2%	17.7%
τ_1 / ns	0.48 (22%)	0.60 (24%)
τ_2 / ns	4.75 (78%)	4.65 (76%)
$\tau_{average}$ / ns	3.81	3.68

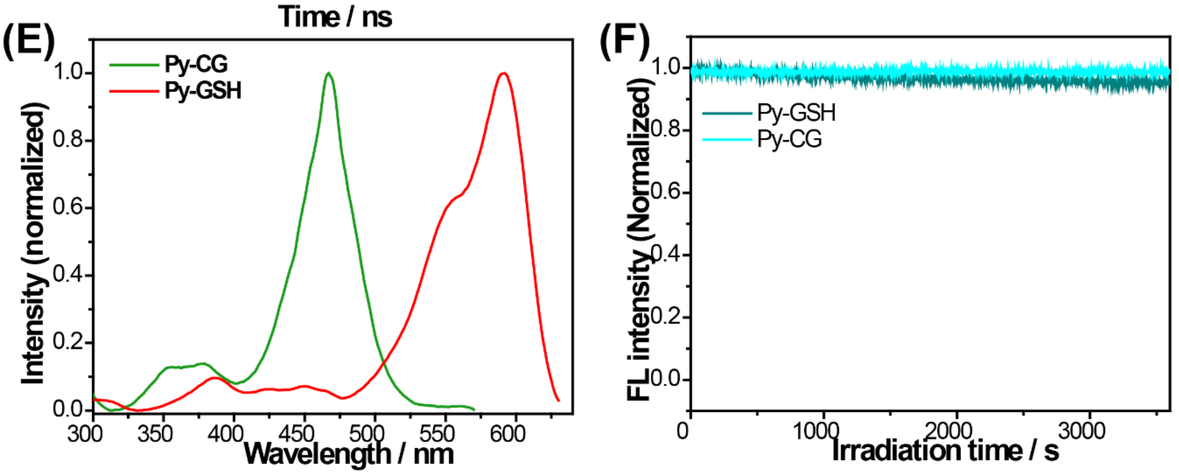

Figure S4. The Quantum yield of Py-GSH (5 μM) in the presence (A) and absence (B) of GGT(50 mU/mL, pH 7.4 PBS). (C) Fluorescence decay and (D) Photophysical properties of Py-GSH in an aqueous solutions incubated with 0 and 50 mU/mL GGT, respectiely. (E) Excitation spectra of Py-GSH in PBS at 650 nm and Py-CG in PBS at 550 nm. (F) Photostability of Py-GSH and Py-CG in PBS. Excitation power: 1 mW / cm^2. Ex = 488 nm.

To measure the quantum yield of Py-GSH, the reference fluorophore is RhB in EtOH (QY = 0.65), Ex = 550 nm; to measure the quantum yield of Py-GSH in the prescence of GGT, the reference fluorophore is fluorescien in pH 7.4 PBS (QY = 0.85), Ex = 470 nm. The quantum yield was calculated in the following manner.

$$\phi = \phi_{ref} \times (n^2_{sample}/n^2_{ref})(I_{sample}/A_{sample})(A_{ref}/I_{ref})$$

Difference concentrations at or below OD 0.1 were measured and the integrated fluorescence was plotted against absorbance for every fluorescent molecular. Comparison of the slopes led to the determination of the quantum yield of Py-GSH and the product after GGT incubation.

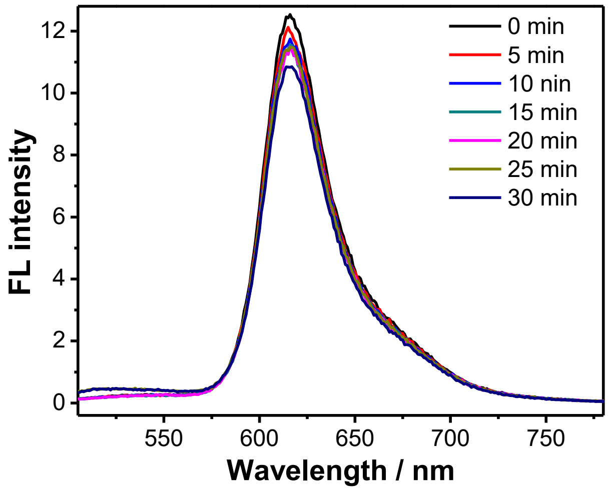

Figure S5. Time dependent fluorescence spectrum of Py-GSH (5 μM) in the prescence of the mixture of GGT and the inhibitor, acivicin. (Ex = 488 nm).

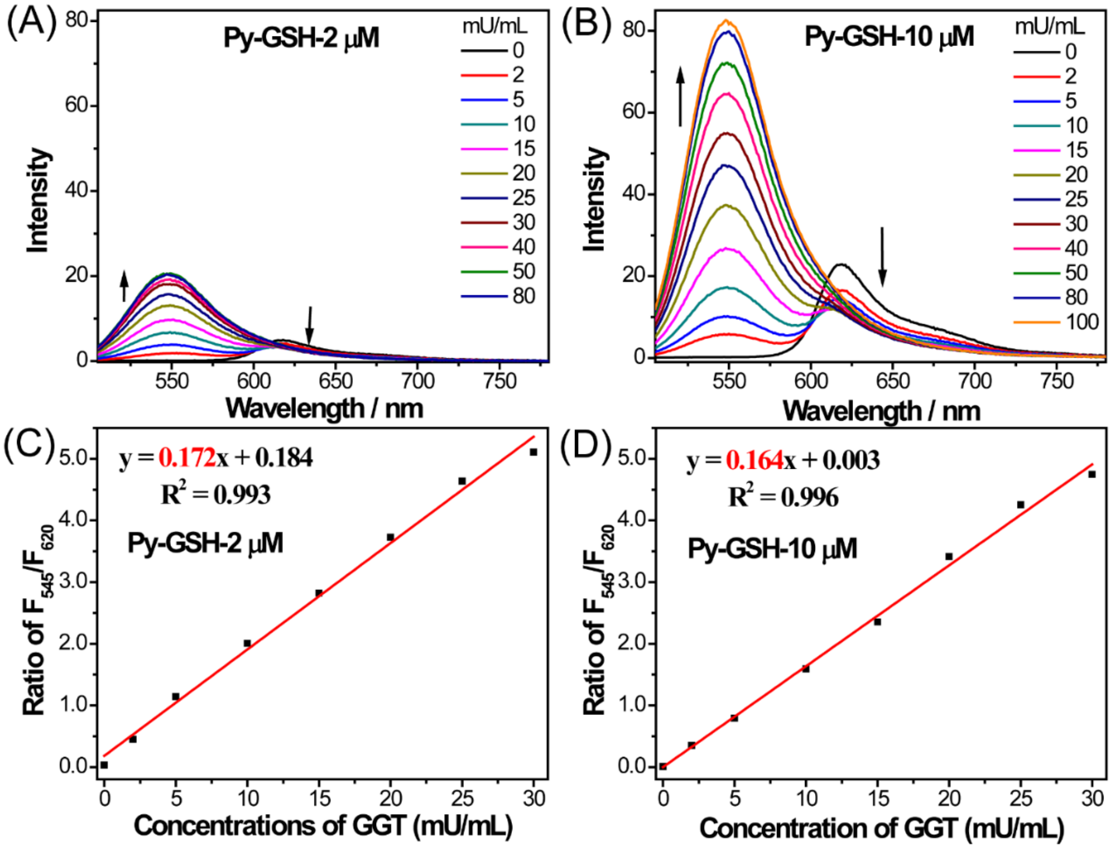

Figure S6. The emission spectra of 2 μM (A) and 10 μM (B) Py-GSH in the presence of different amount of GGT; plots of the fluorescence intensity ratio from 545 nm to 620 nm (F_{545}/F_{620}) as a function of GGT concentration of (C) 2 μM and (D) 10 μM Py-GSH.

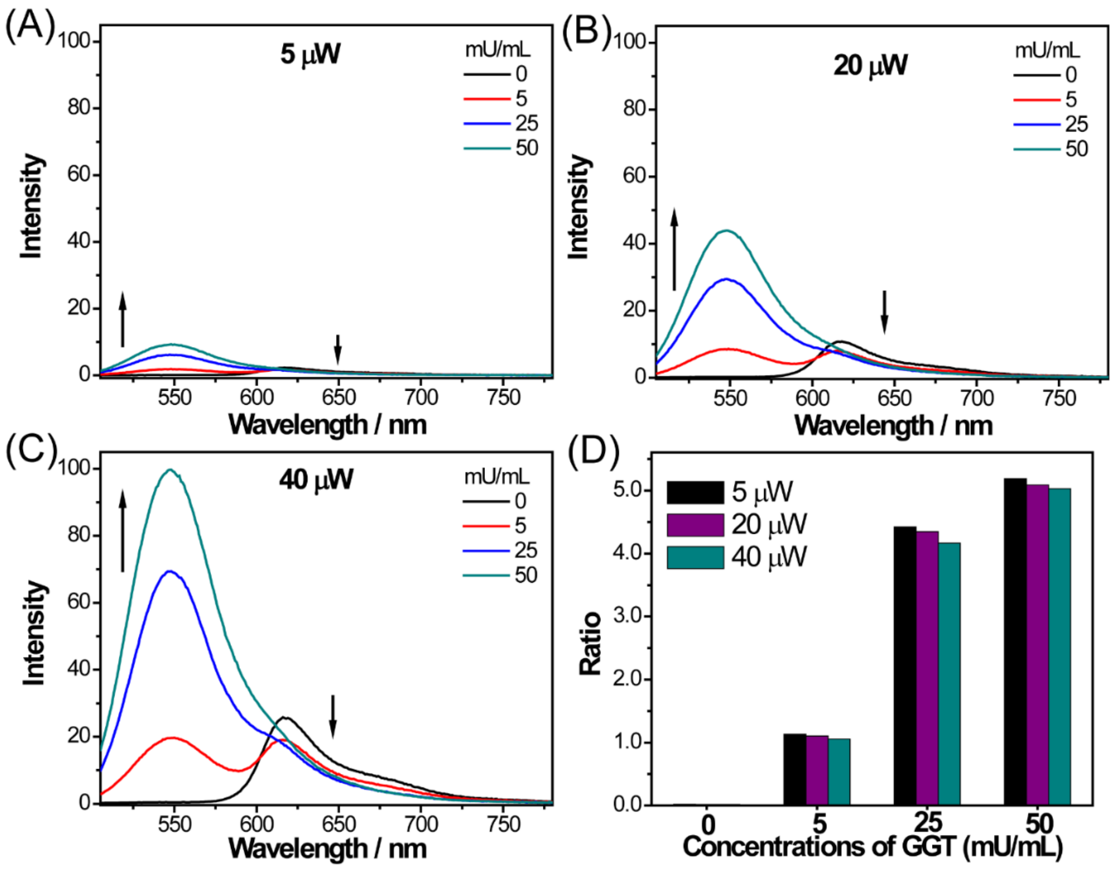

Figure S7. The emission spectrum of Py-GSH (5 μM) in the prescence of different amount of GGT for 30 min under the excitation slits as (A) 5 μW/cm^2, (B) 20 μW/cm^2 and (C) 40 μW/cm^2; (D) effect of the different excitation slits for F_{545}/F_{620}, fluorescence intensity at 620 nm and 545 nm of 5μM Py-GSH after treated with 0, 5, 25, 50 mU/mL GGT for 30 min, respectively.

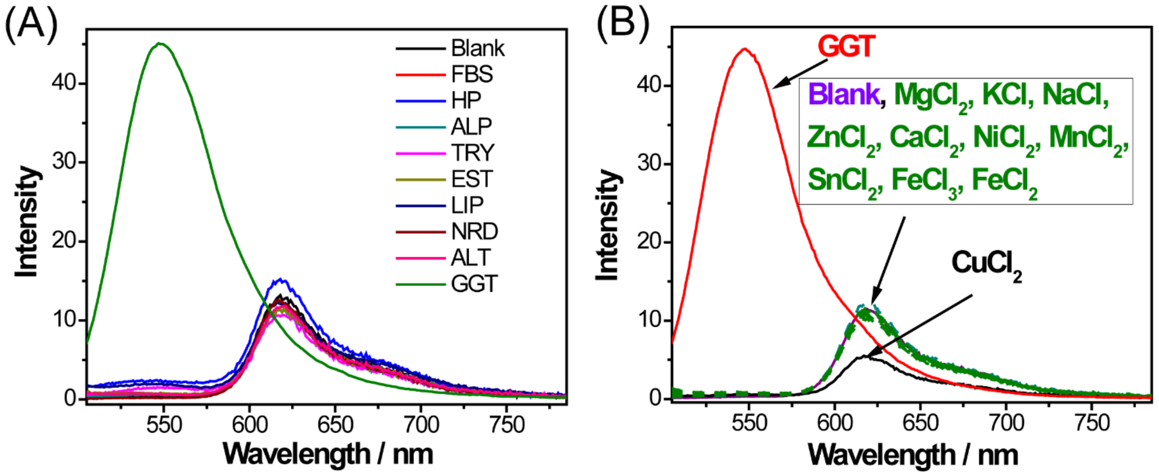

Figure. S8. (A) Fluorescence spectrum in the presence of fetal 10 % bovine serum (FBS) , 5% human plasma (HP), 0.5 U/mL alkaline phosphatase (ALP), 0.2 U/mL trypsase (TRY), 0.5 U/mL esterase (EST), 0.5 U/mL lipase (LIP), 0.1 U/mL reductase (NRD), 0.2 U/mL alanine transaminase (ALT) and 0.05 U/mL γ-glutamyltranspeptidase (GGT); (B) fluorescence spectrum in the presence of different biological ions (NaCl-10 mM, KCl-10 mM, $MgCl_2$-2.5 mM, $CaCl_2$-2.5 mM, $ZnCl_2$-1 mM, $NiCl_2$-0.2 mM, $MnCl_2$-0.1 mM, $SnCl_2$-0.1 mM, $FeCl_2$-0.1 mM, $FeCl_3$-0.1 mM, $CuCl_2$-0.1 mM) and 0.05 U/mL GGT. λ_{ex}=488 nm.

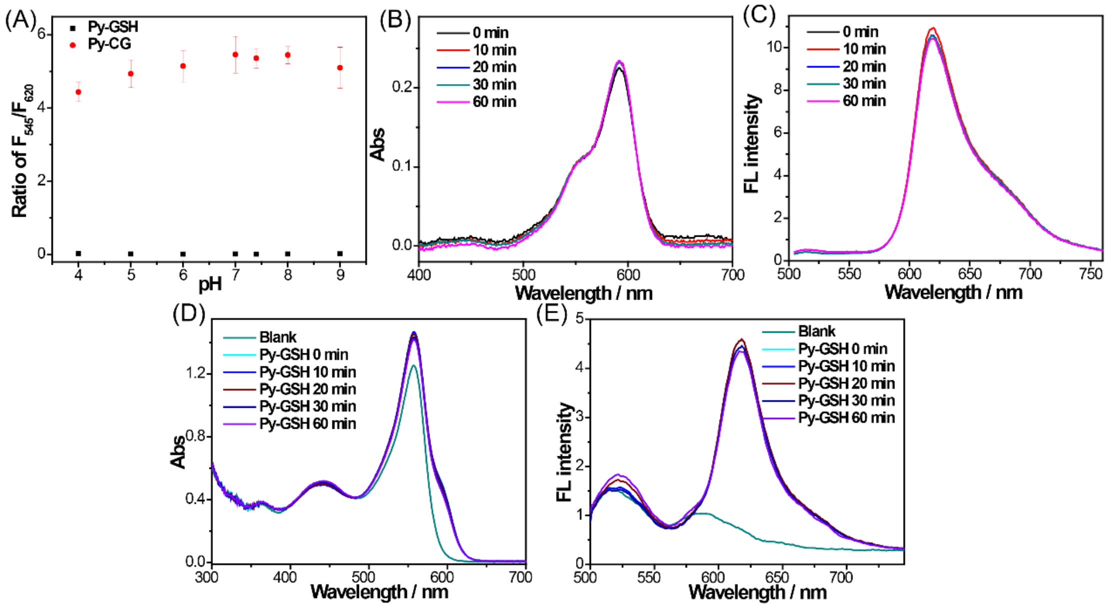

Figure S9. (A) Effect of pH on the emission ratio of F_{545}/F_{620} with Py-GSH and Py-CG. Time dependent absorption (B) and emission (C) spectra of Py-GSH in PBS. Time dependent

absorption (D) and emission (E) spectra of Py-GSH in DMEM (cell culture medium). λ_{ex}=488 nm.

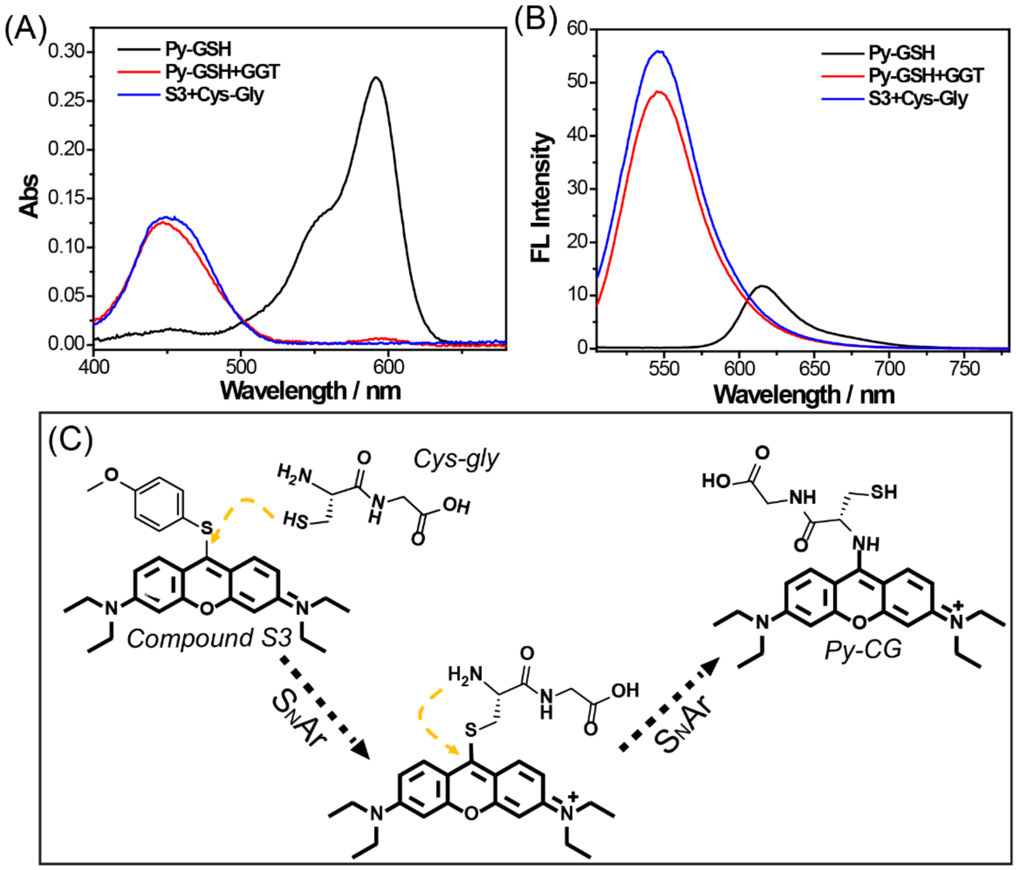

Figure S10.. (A) Absorption and (B) fluorescence spectral of Py-GSH (5μM), 50 mU/mL GGT incubated Py-GSH (5 μM) for 20 min and 20 μM Cys-Gly incubated S3 (5 μM) for 20 min, respectively. (Ex=488 nm); (c) The reaction of compound S3 and Cys-gly.

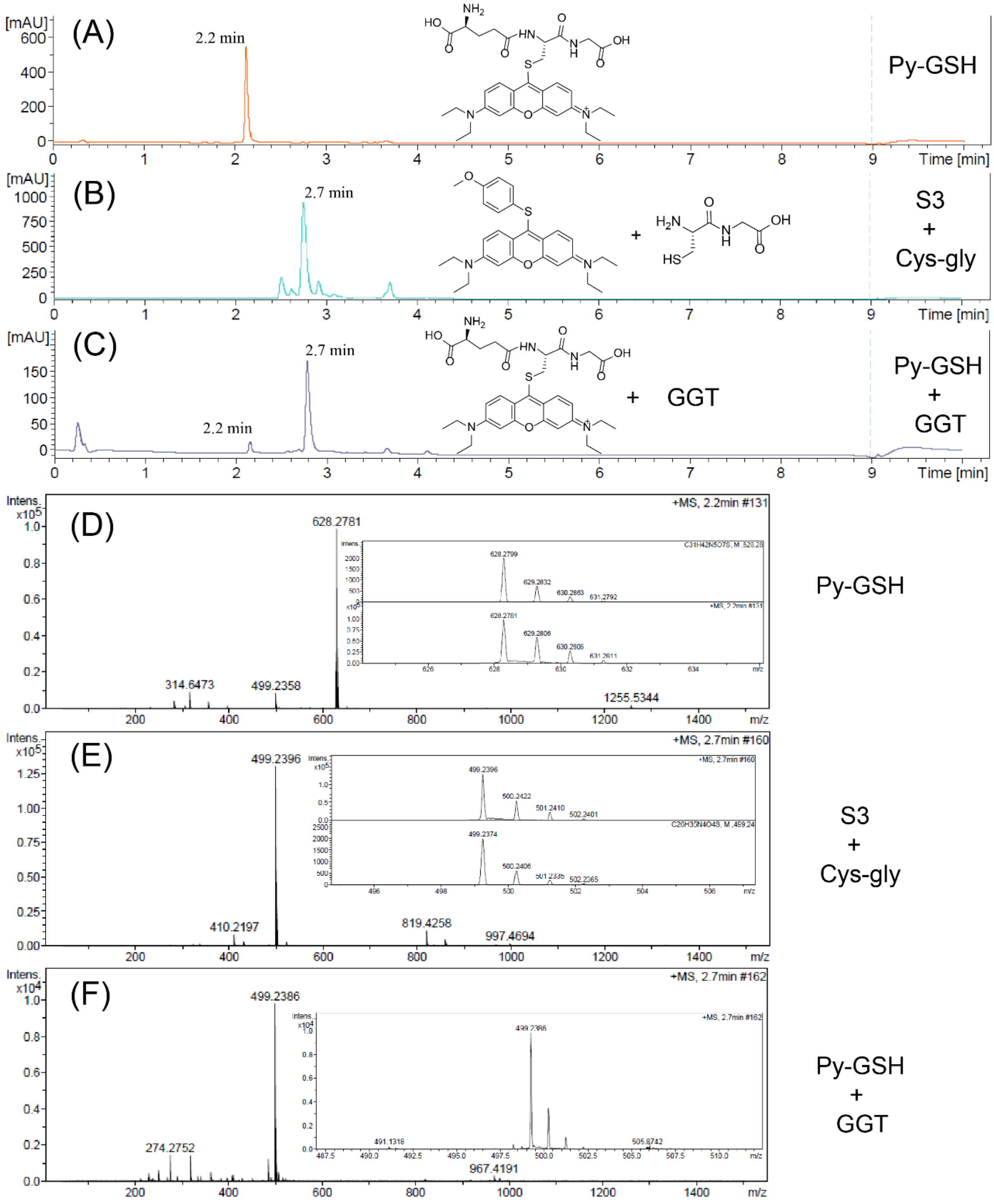

Fig S11. A-C) Typical HPLC chromatogram and D-F) the corresponding mass spectra of the Py-GSH (10 μM), 100 mU/mL GGT incubated Py-GSH (10 μM) for 20 min and 100 μM Cys-Gly incubated S3 (10 μM) for 20 min. Peaks in the chromatograms were detected by monitoring the absorption at 254 nm. The mobile phase was 10/90 CH_3CN/water at a flow of 1 mL/min.

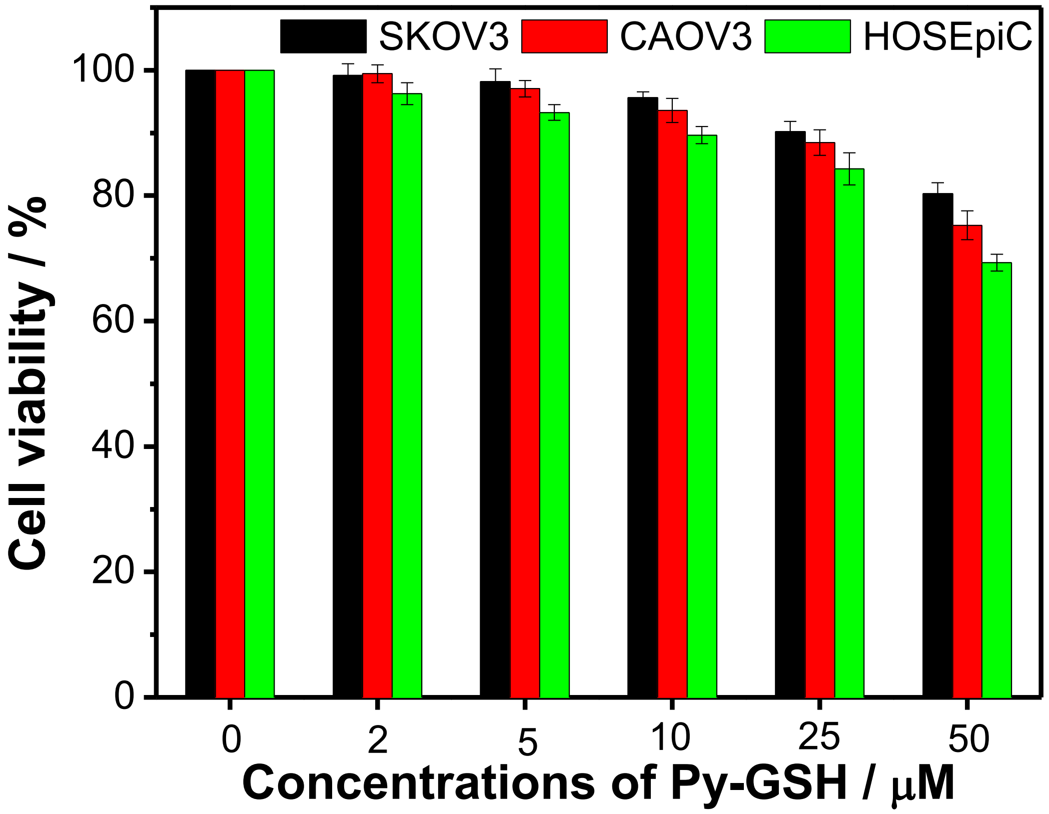

Figure S12. Dose-response curves for cell viability of SKOV3, CAOV3, HOSEpiC cells treated with Py-GSH by using a typical MTT assay. Error bars correspond to standard deviations from three separate measurements.

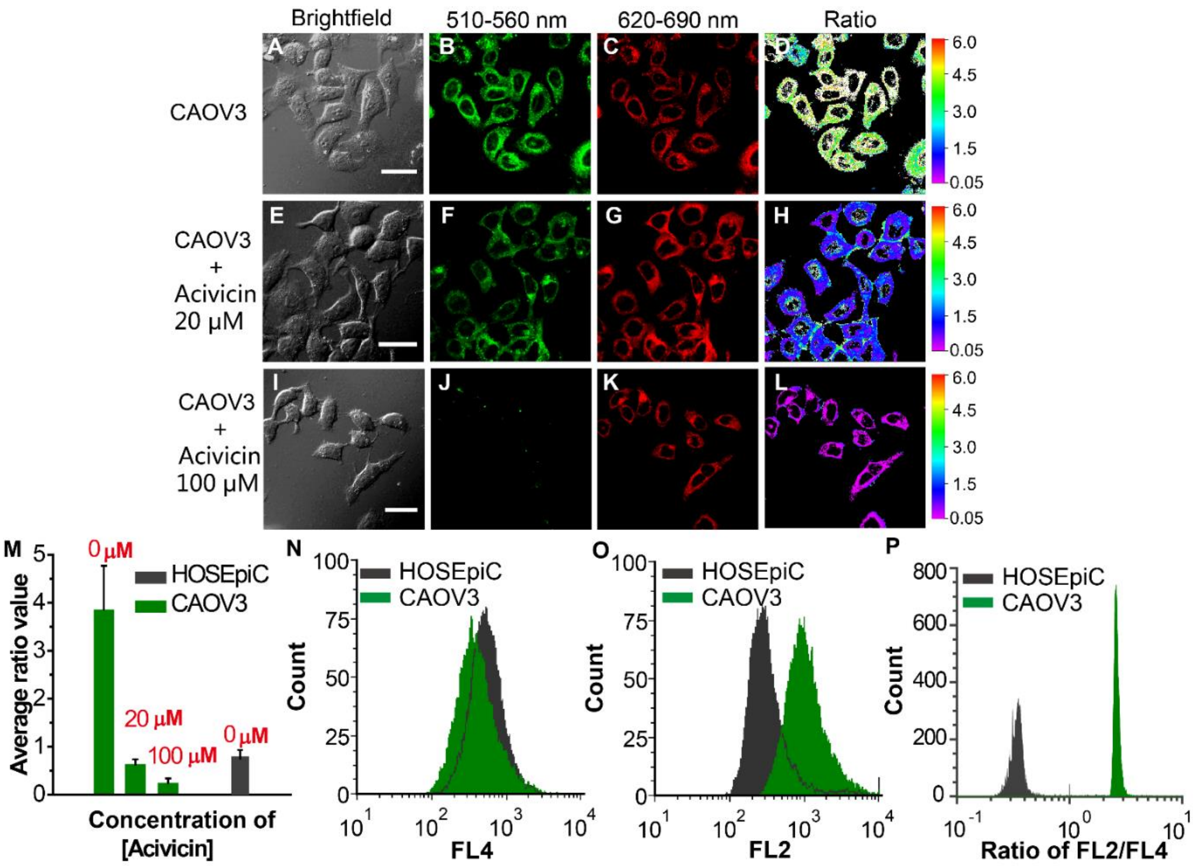

Figure S13. Fluorescence images of CAOV3 (A-D) and acivicin pretreated CAOV3 (E-L) upon incubated with Py-GSH (5 μM) for 30 min. The emission signal of probe were collected at 510-560 nm (green channel) and 620-690 nm (red channel), respectively. The ratio image generated from green to red channel. CAOV3 were pretreated with acivicin (20 μM, 100 μM) for 30 min then incubated with Py-GSH (5 μM). Scale bar, 30 μm.(Q) Quantification of average ratio value in images of CAOV3 and acivicin treated CAOV3. (N-P) Flow cytometric analysis of CAOV3 and HOSEpiC cells after incubated with Py-GSH (5 μM) for 30 min. FL2: 560±15 nm, FL4: 675±15 nm. Ex = 488 nm

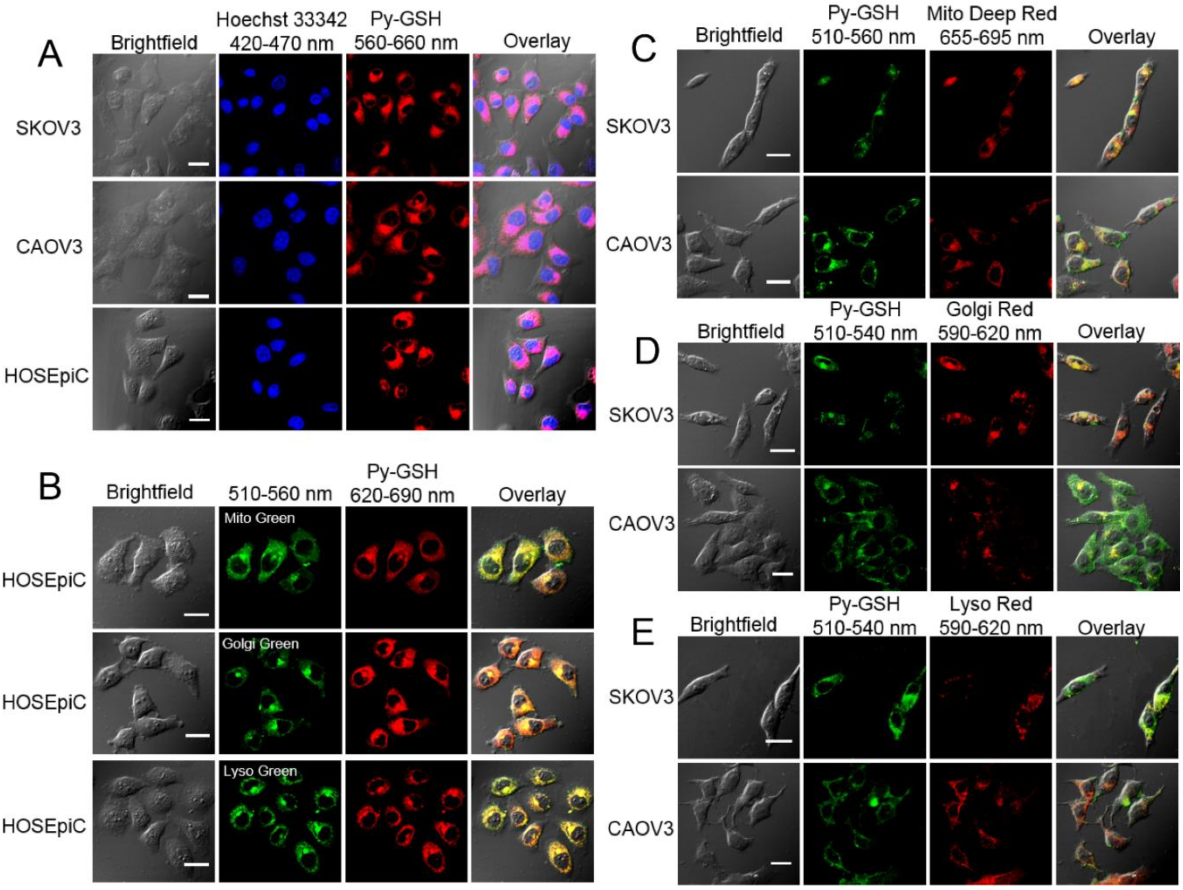

Figure S14. Co-localization of Py-GSH (5 μM) and organelle specific dyes in SKOV3, CAOV3, HOSEpiC. (A) Costaining of Py-GSH (collected at 560-660 nm,) and Hoechst 33342 (collected at 420-470 nm, Ex = 405 nm) in SKOV3, CAOV3, HOSEpiC; (B) costaining of Py-GSH (collected at 620-690 nm) and mito-tracker green, golgi-tracker green and lyso-tracker green (collected at 510-560 nm) in HOSEpiC; (C) costaining of Py-GSH (collected at 510-560 nm) and mito-tracker deep red (collected at 655-695 nm) in SKOV3, CAOV3; (D) costaining of Py-GSH (collected at 510-540 nm) and golgi-tracker red (collected at 590-620 nm) in SKOV3, CAOV3; (E) co-staining of Py-GSH (collected at 510-540 nm) and lysotracker red (collected at 590-620 nm) in SKOV3, CAOV3. Cells were incubated with Py-GSH for 30 min and then co-stained with 2 μM organelle specific dyes for 10 min. Ex=488 nm.

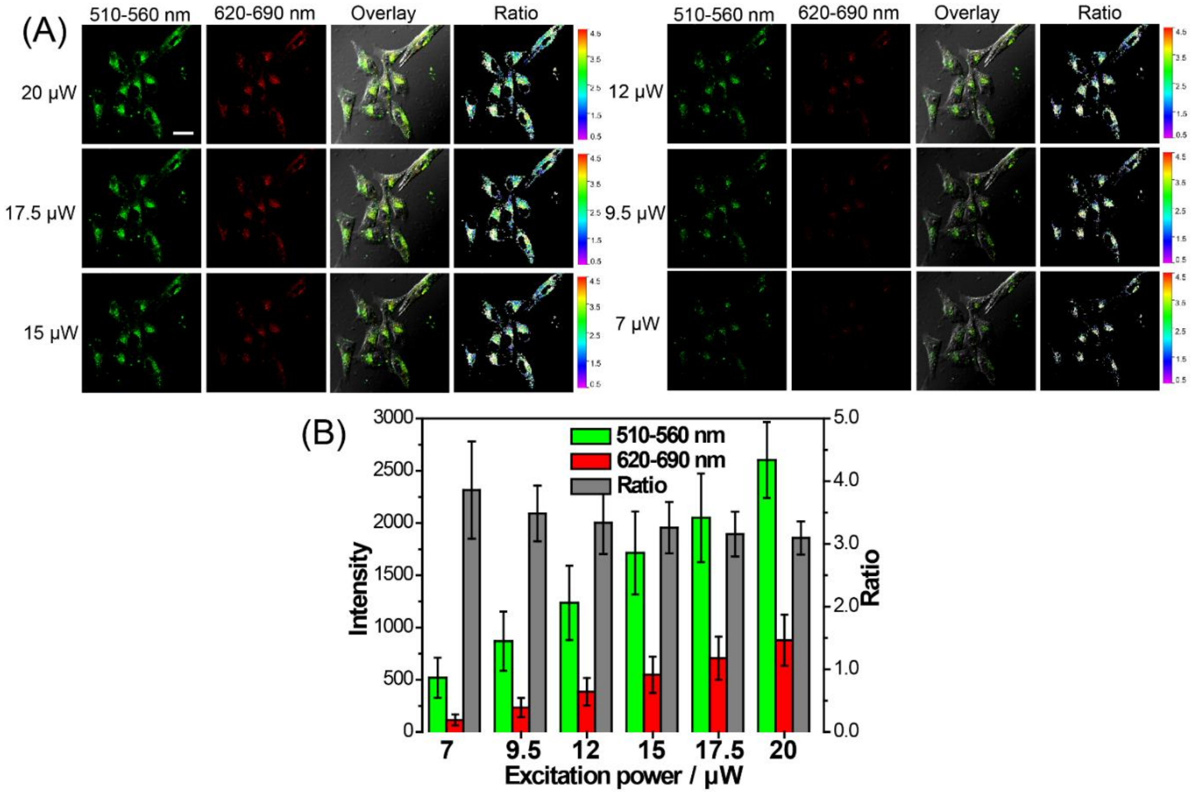

Figure S15. (A) Fluorescence confocal image of SKOV3 cells incubated with Py-GSH (5 μM) 30 min under different excitation power. The fluorescence image were separately collected at 510-560 nm and 620-690 nm, ratio of emission intensity at 510-560 nm to that at 620-690 nm was shown. Ex = 488 nm. Scale bar: 30 μm; (B) The effect of excitation power toward the average intensity of the collected fluorescence signal at 510-560 nm, 620-690 nm and the average ratio value to the imaging areas.

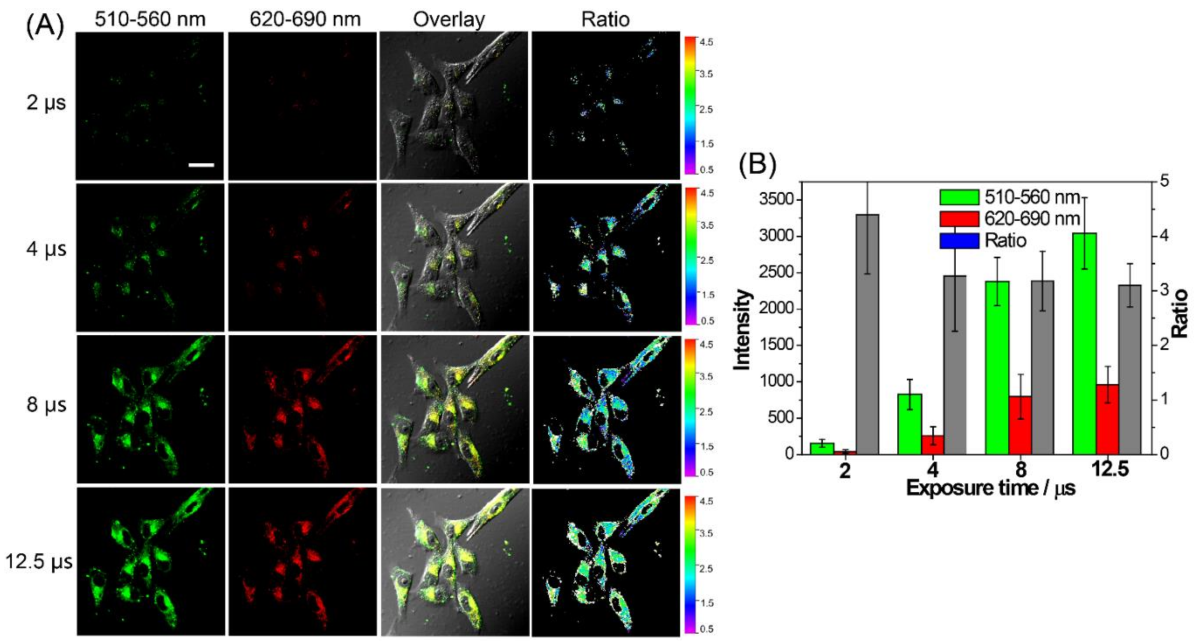

Figure S16. (A) Fluorescence confocal image of SKOV3 cells incubated with Py-GSH (5 μM) under different exposure time. The fluorescence image were separately collected at 510-560 nm and 620-690 nm, Ratio of emission intensity at 510-560 nm to that at 620-690 nm was shown, Ex = 488 nm. Scale bar: 30 μm; (B) The effect of exposure time toward the average intensity of the collected fluorescence signal at 510-560 nm, 620-690 nm and the average ratio value to the imaging areas.

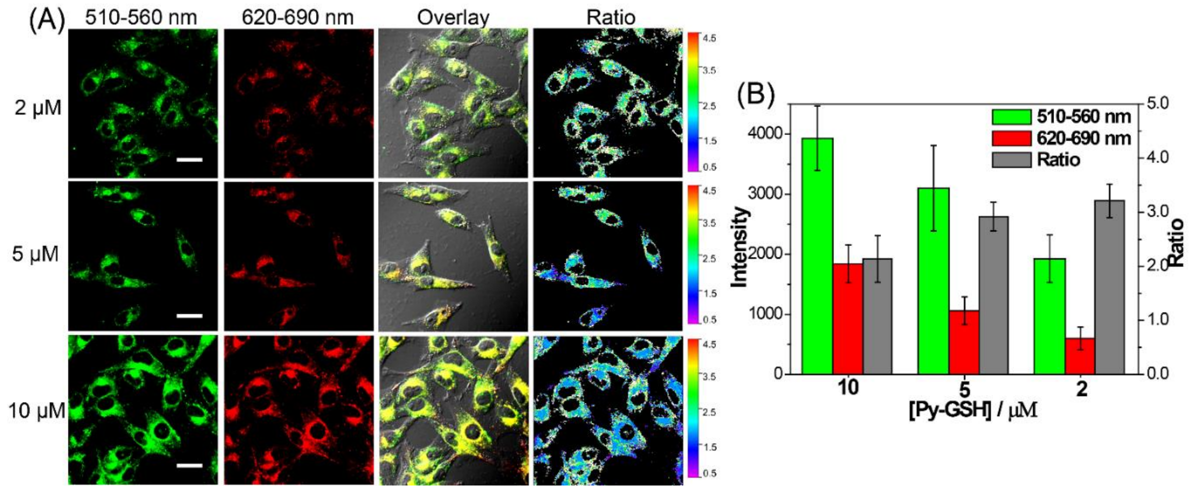

Figure S17. (A) Fluorescence confocal image of SKOV3 cells incubated with different concentration of Py-GSH. The fluorescence image were separately collected at 510-560 nm and 620-690 nm, Ratio of emission intensity at 510-560 nm to that at 620-690 nm was shown. Ex = 488 nm. Scale bars, 30 μm; (B) The effect of the incubated concentration of Py-GSH toward the average intensity of the collected fluorescence signal at 510-560 nm, 620-690 nm and the average ratio value to the imaging areas.

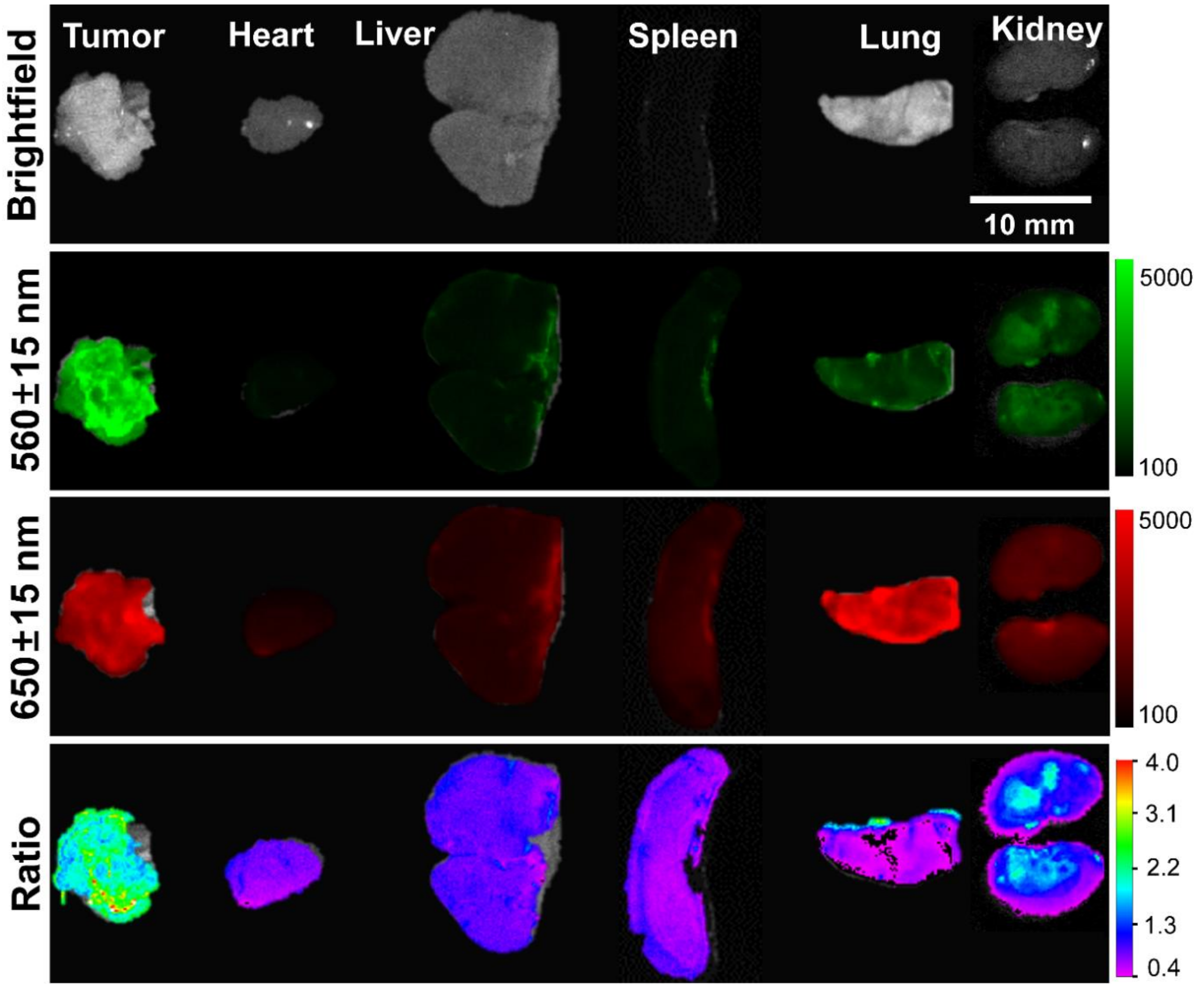

Figure S18. Fluorescence images of the main internal organs and tumor tissues of tumor-bearing mice after stain with 10 μM Py-GSH saline for 10 min. In fluorescence imaging, the emission channel at 560±15 nm (Green channel) and 650±15 nm (Red channel) were collected. In ratiometric imaging, the ratio of emission intensity at 560±15 nm to that at 650±15 nm was chosen as the detected signal. Ex=490 nm

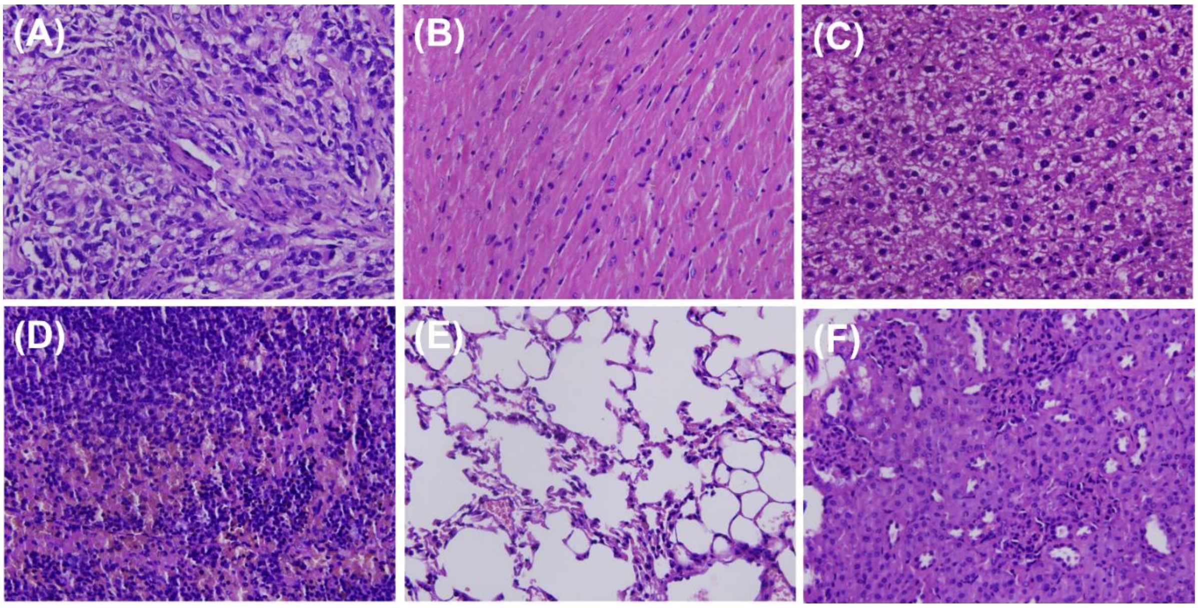

Figure S19. H&E staining specimen of subcutaneous tumor (SKOV3) mice tissues as shown in Figure S19. (A) Tumor, (B) heart; (C) liver; (D) spleen; (E) lung; (F) kidney

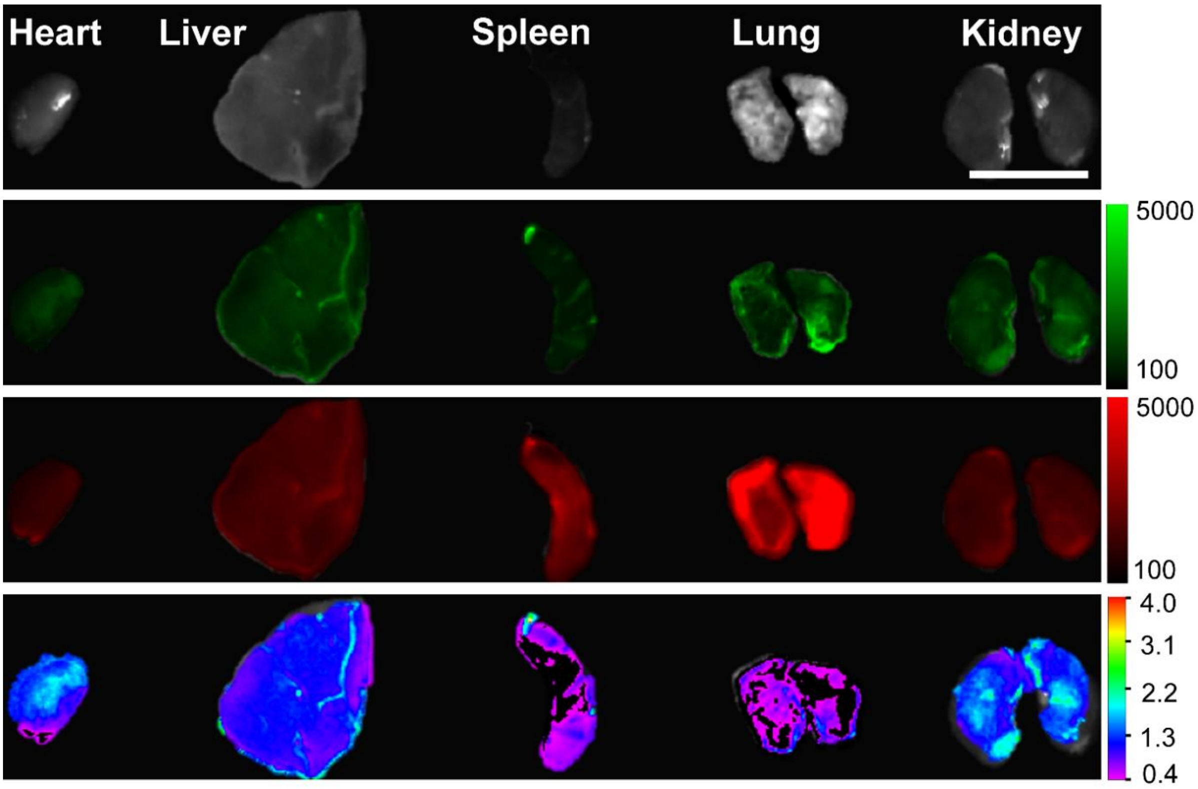

Figure S20. Fluorescence images of the main internal organs from normal mice after stain with 10 μM Py-GSH saline for 10 min. In fluorescence imaging, the emission channel at 560±15 nm (Green channel) and 650±15 nm (Red channel) were collected. In ratiometric imaging, the ratio of emission intensity at 560±15 nm to that at 650±15 nm was chosen as the detected signal. Ex=490 nm.

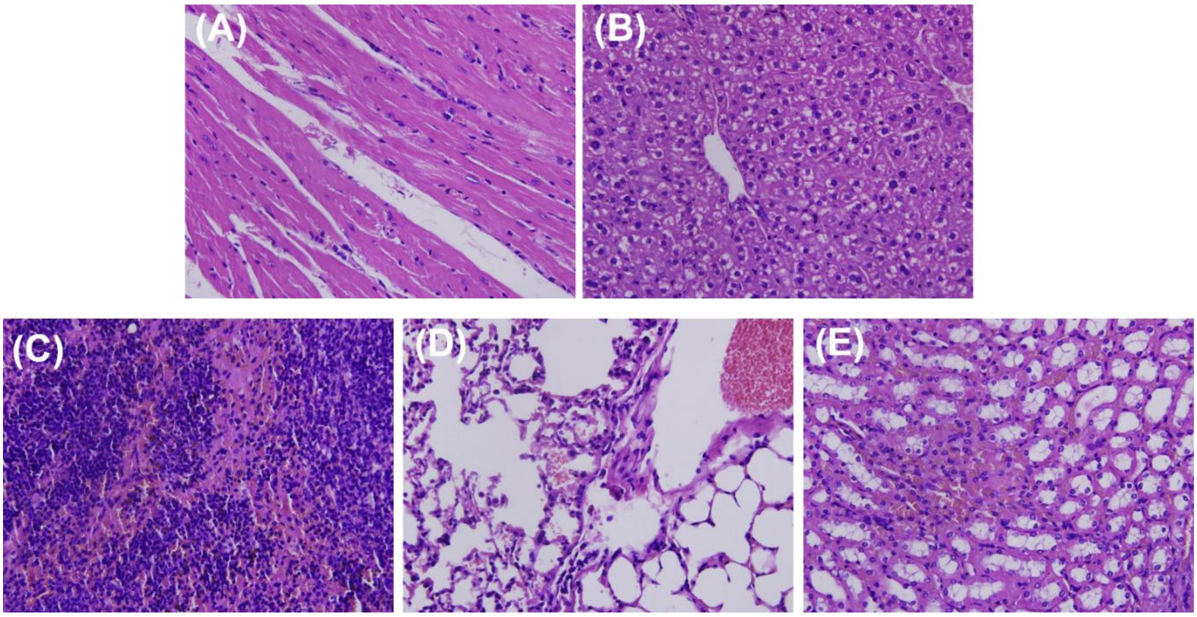

Figure S21. H&E staining specimen of normal mice tissues as shown in Figure S21. (A) Heart; (B) liver; (C) spleen; (D) lung; (E) kidney.

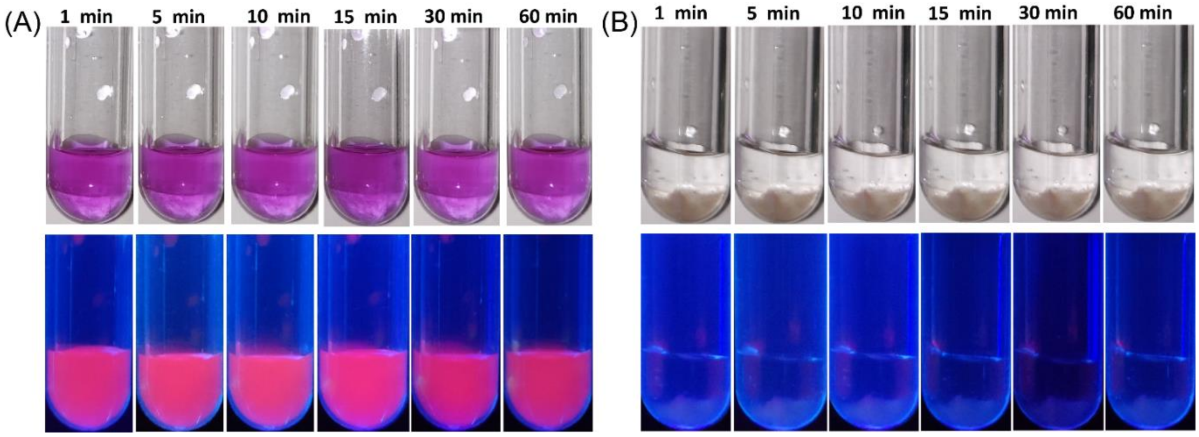

Figure S22. Time-dependent photo images of control groups. (A) 10 μM Py-GSH saline for 60 min, (B) tumor tissue treated with saline for 60 min. Excitation source, 365 nm lamp

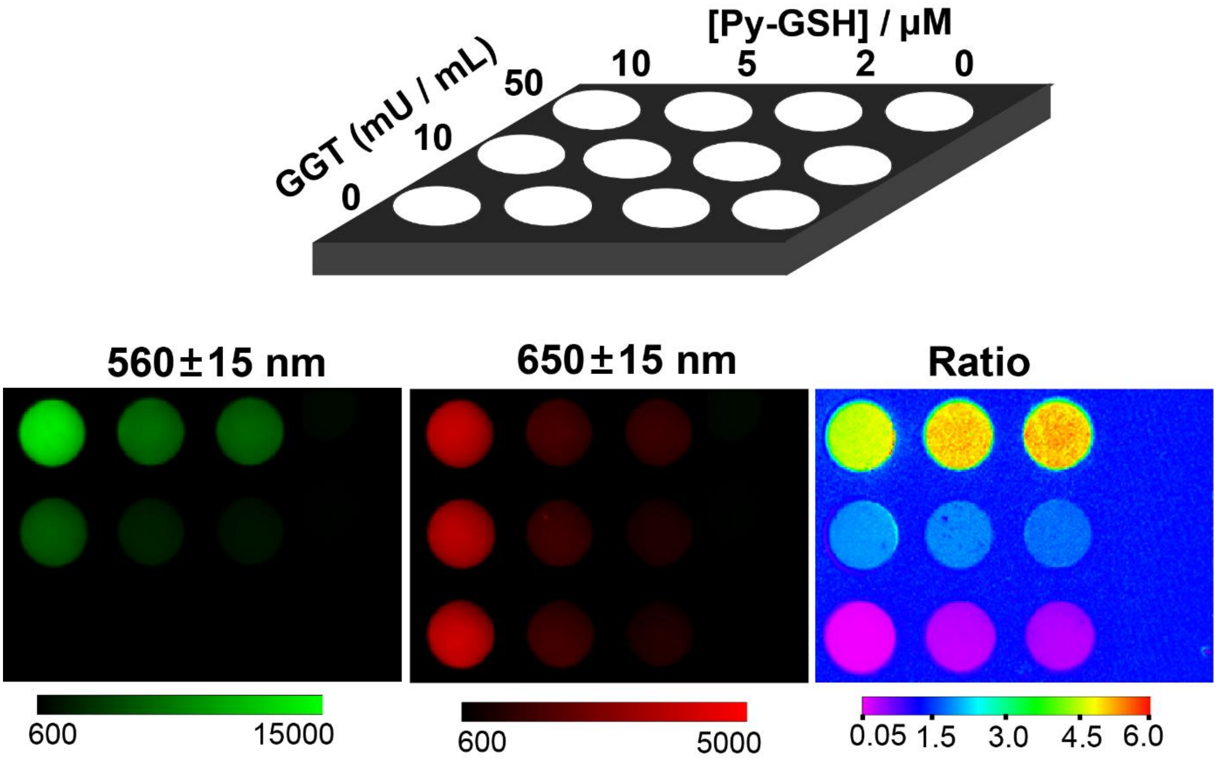

Figure S23. Fluorescence imaging and ratiometric fluorescence imaging of different concentrations of Py-GSH solutions after the solutions incubated with different concentrations of GGT for 30 min (pH 7.4 PBS, 37 ℃). Emission channel at 560±15 nm (Green channel) and 650±15 nm (Red channel) were collected. The ratio signal was calculated from the emission intensity at 560±15 nm to that at 650±15 nm.

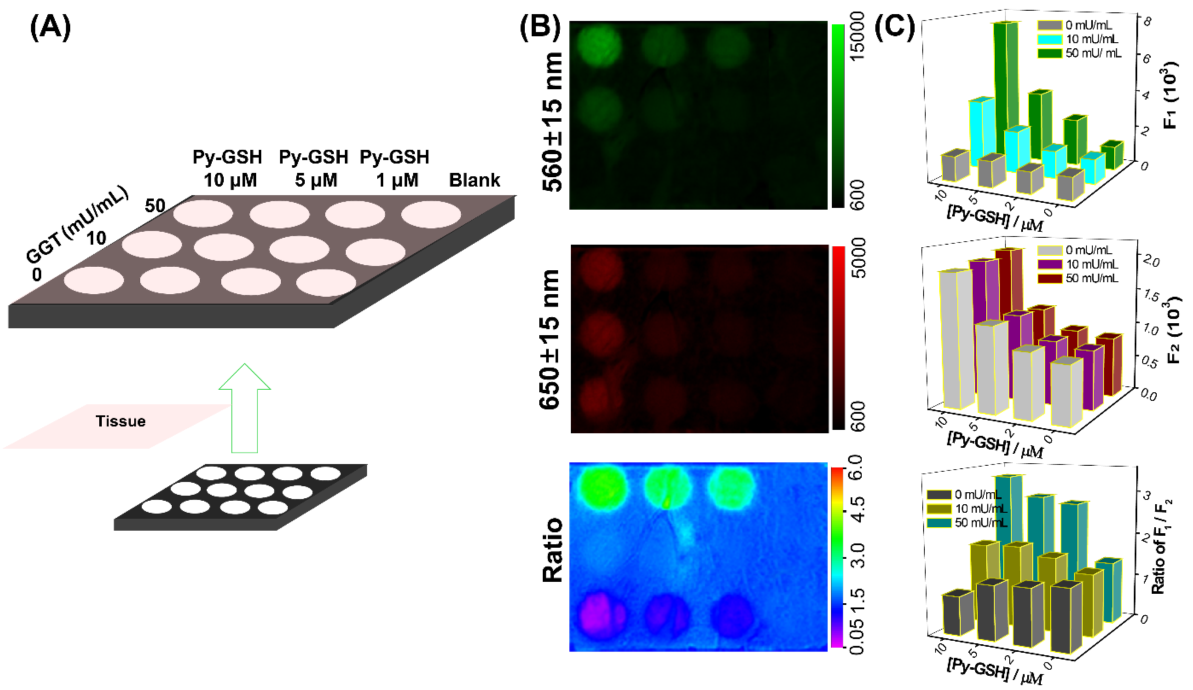

Figure S24. (A) Simplified diagram depicting the experimental setup of the concentration and tissue interference experiment. (B) Fluorescence imaging and ratiometric fluorescence imaging of different concentrations of Py-GSH solutions after the solutions incubated with different concentrations of GGT for 30 min (pH 7.4 PBS, 37 ^{o}C) under the cover of 1 mm pork tissue. Emission channel at 560±15 nm (Green channel) and 650±15 nm (Red channel) were collected. The ratio signal was calculated from the emission intensity at 560±15 nm to that at 650±15 nm. (C) The average emission intensity of different signal collecting channel and average ratio value of every well. Ex=490 nm

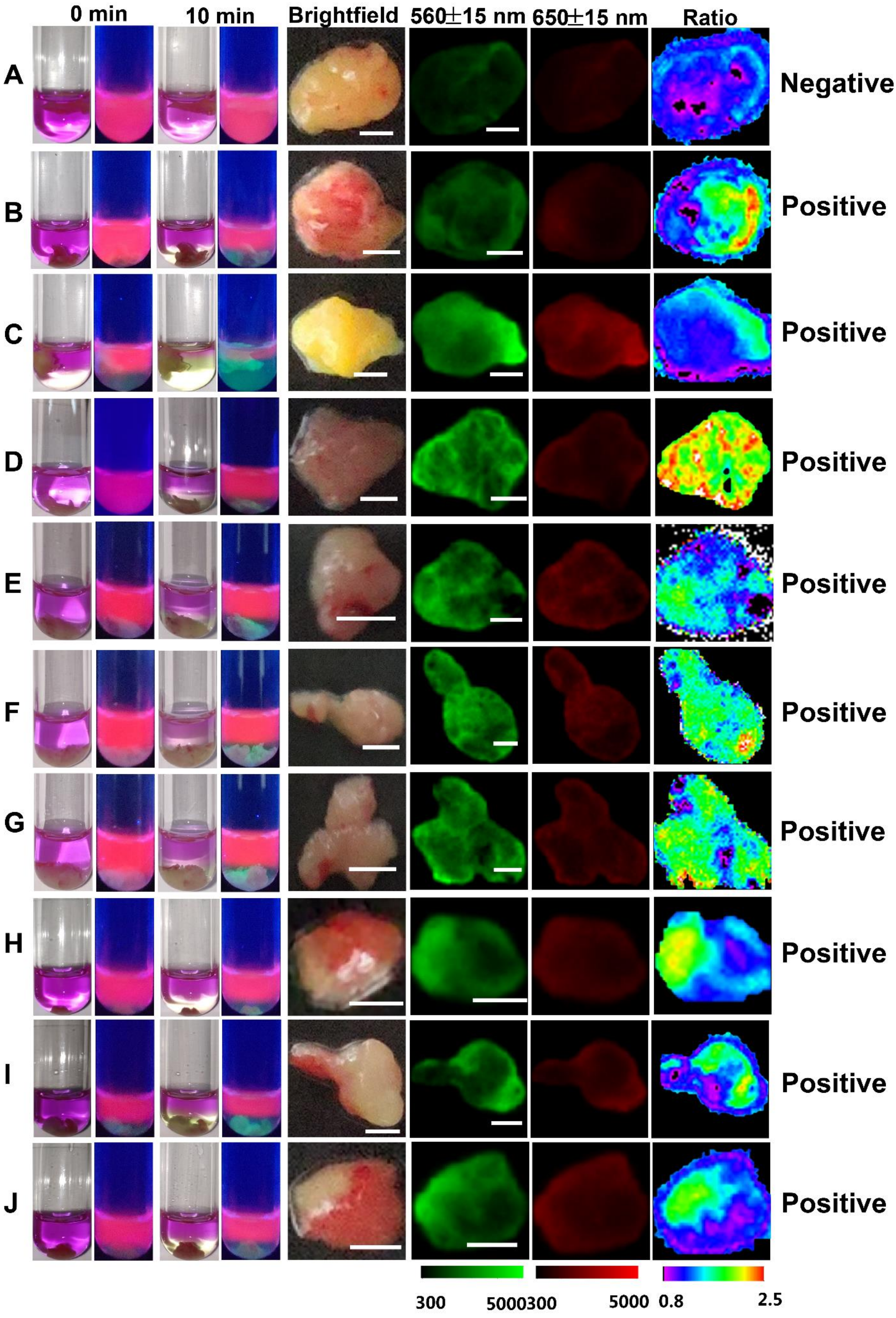
0 min
10 min
Brightfield
560±15 nm
650±15 nm
Ratio
A
Negative
B
Positive
C
Positive
D
Positive
E
Positive
F
Positive
G
Positive
H
Positive
I
Positive
J
Positive
300
5000
300
5000
0.8
2.5

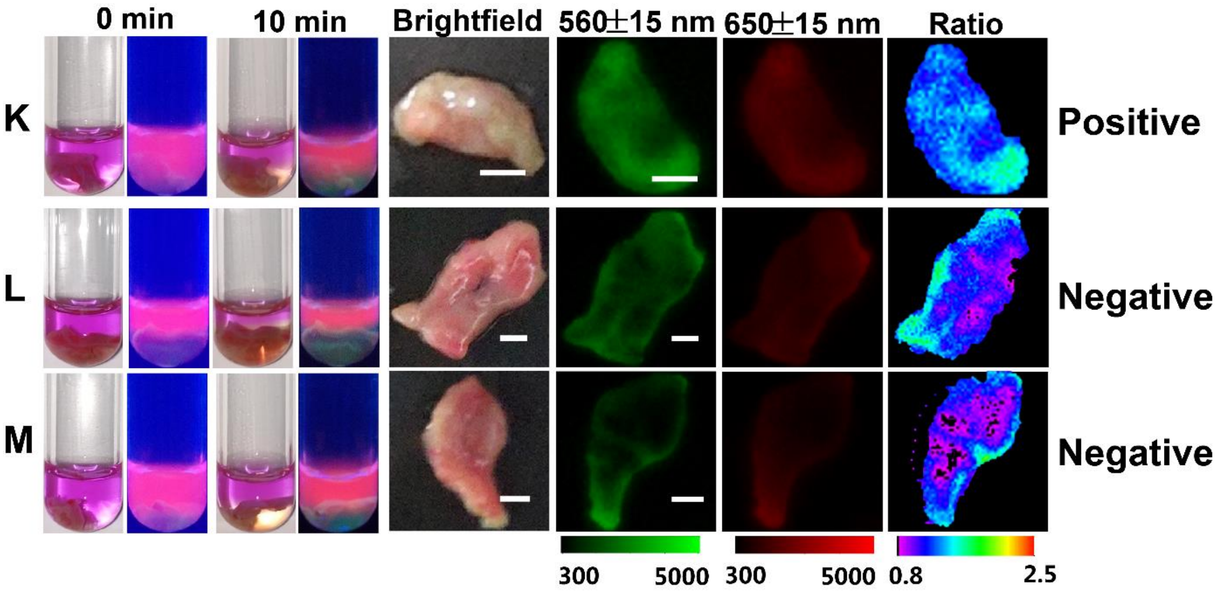

Figure S25. Fluorescence images of the human tissues after stain with 10 μM Py-GSH saline for 10 min. Tumor tissue (A-J), normal tissue (K-M). In fluorescence tissue imaging, the emission channel at 560±15 nm (Green channel) and 650±15 nm (Red channel) were collected. In ratiometric imaging, the ratio of emission intensity at 560±15 nm to that at 650±15 nm was chosen as the detected signal. Ex = 490 nm. Scale bar, 2 mm.

Glu-Dye	Dye	Response Type	Km μM	LOD mU/L	Δλ Abs (nm)	Δλ Em (nm)	Reference
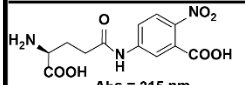 Abs = 315 nm<br>Em No emission	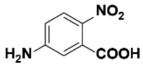 Abs = 380 nm<br>Em No emission	Colorimetric	28	N.D.	65	N.D.	IFCC
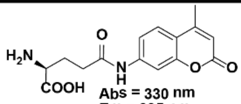 Abs = 330 nm<br>Em = 395 nm	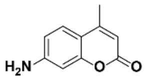 Abs = 350 nm<br>Em = 440 nm	Turn on<br>Ex = 370 nm	410	0.05 μM	20	45	Anal. Biochem. 1979, 100, 136
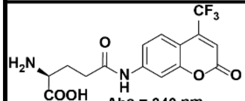 Abs = 340 nm<br>Em = 445 nm	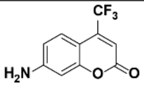 Abs = 380 nm<br>Em = 500 nm	Turn on<br>Ex = 400 nm	900	N.D.	20	55	Anal. Biochem. 1996, 233, 71
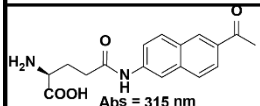 Abs = 315 nm<br>Em = 445 nm	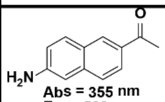 Abs = 355 nm<br>Em = 500 nm	Ratiometric<br>Ex = 355 nm (730 nm)	9.8	300	40	55	New J. Chem. 2018, 42, 5403
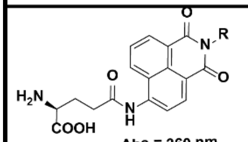 Abs = 360 nm<br>Em = 475 nm	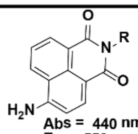 Abs = 440 nm<br>Em = 550 nm	Ratiometric<br>Ex = 408 nm	N.D.	760	80	75	Chem. Commun. 2014, 50, 3417
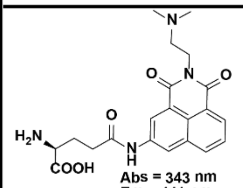 Abs = 343 nm<br>Em = 441 nm	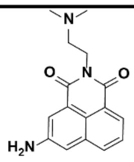 Abs = 354 nm<br>Em = 531 nm	Ratiometric<br>Ex = 417 nm (800 nm)	N.D.	182	11	90	Chem. Commun. 2016, 52, 6308
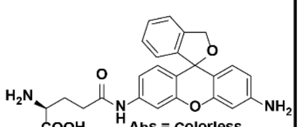 Abs = Colorless<br>Em = n.d.	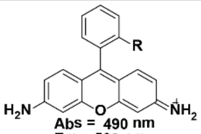 Abs = 490 nm<br>Em = 520 nm	Turn on<br>Ex = 496 nm	145	N.D.	N.D.	N.D.	Sci. Transl. Med. 2011, 3, 110ra119
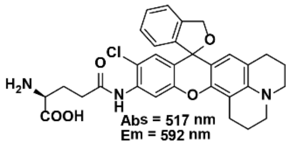 Abs = 517 nm<br>Em = 592 nm	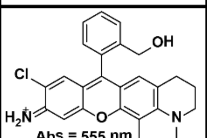 Abs = 555 nm<br>Em = 582 nm	Turn on<br>Ex = 550 nm	45.3	N.D.	38	10	Chem. Eur. J. 2016, 22, 1696-1703
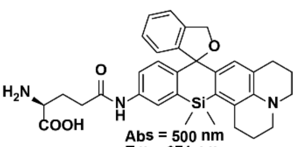 Abs = 500 nm<br>Em = 671 nm	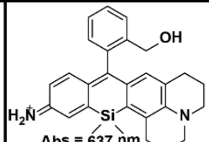 Abs = 637 nm<br>Em = 662 nm	Turn on<br>Ex = 637 nm	N.D.	N.D.	137	9	Bioconjugate Chem. 2018, 29, 241-244

Table S1. Chemical structure of reported GGT-activatable fluorescence probes and their photophysical properties.

Glu-Dye	Dye	Response Type	Km μM	LOD mU/L	Δλ Abs (nm)	Δλ Em (nm)	Reference
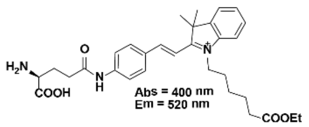 Abs = 400 nm Em = 520 nm	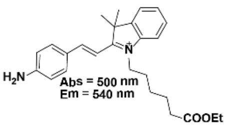 Abs = 500 nm Em = 540 nm	**Turn on** Ex = 440 nm	**N.D.**	**160**	**100**	**20**	**Chem. Commun., 2016, 52, 10400**
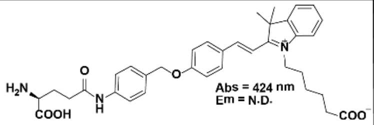 Abs = 424 nm Em = N.D.	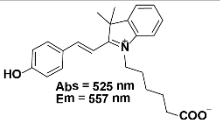 Abs = 525 nm Em = 557 nm	**Ratiometric** Ex = 460 nm	**N.D.**	**150**	**101**	**N.D.**	**ACS Sens. 2016, 1, 579**
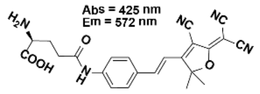 Abs = 425 nm Em = 572 nm	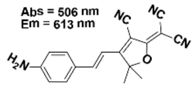 Abs = 506 nm Em = 613 nm	**Turn on** Ex = 506 nm	**11.48**	**37.9**	**81**	**41**	**Biomaterials, 2018, 179, 1**
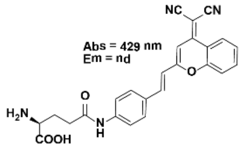 Abs = 429 nm Em = nd	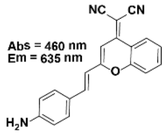 Abs = 460 nm Em = 635 nm	**Ratiometric** Ex = 490 nm	**10.27**	**57**	**31**	**N.D.**	**Biomaterials 2016, 80, 46**
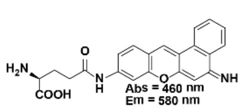 Abs = 460 nm Em = 580 nm	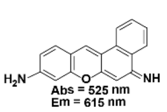 Abs = 525 nm Em = 615 nm	**Turn on Ratiometic** Ex = 585 nm	**7.64**	**5.6**	**65**	**35**	**Anal. Chem. 2015, 87, 8353** **Adv. Funct. Mater. 2018, 28, 1703450**
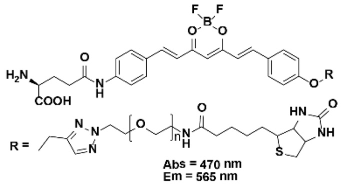 Abs = 470 nm Em = 565 nm	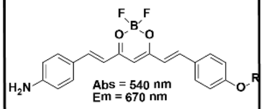 Abs = 540 nm Em = 670 nm	**Dual channel** Ex = 600 nm	**6.62**	**78.5**	**70**	**105**	**Chem. Commun. 2018, 54, 12393**
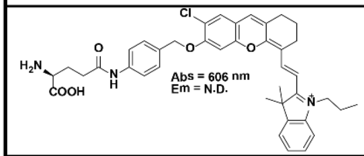 Abs = 606 nm Em = N.D.	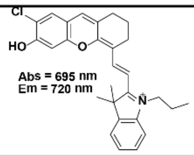 Abs = 695 nm Em = 720 nm	**Turn on** Ex = 680 nm	**1.26**	**3.6**	**89**	**N.D.**	**Chem. Eur. J. 2017, 23. 14778**
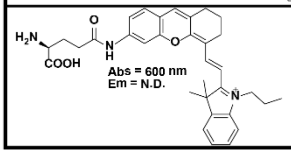 Abs = 600 nm Em = N.D.	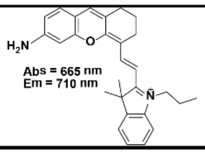 Abs = 665 nm Em = 710 nm	**Turn on** Ex = 680 nm	**28.2**	**1200**	**89**	**N.D.**	**Anal. Bioanal. Chem. 26, 6771**

Table S1. continued

Glu-Dye	Dye	Response Type	Km µM	LOD mU/L	Δλ Abs (nm)	Δλ Em (nm)	Reference
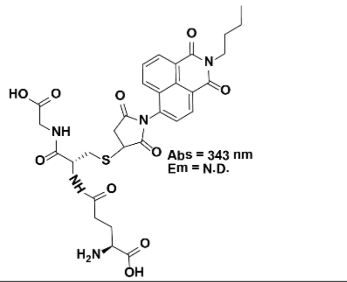	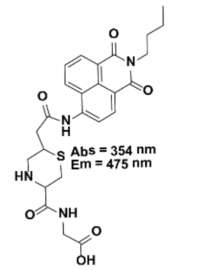	**Turn on** Ex = 345 nm	**17.64**	**210**	**11**	**N.D.**	**Anal. Chem. 2016, 88, 10816**
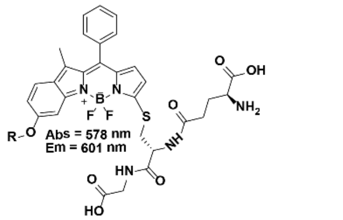	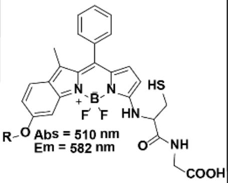	**Ratiometric** Ex = 450 nm	**18.76**	**N.D.**	**68**	**19**	**Angew. Chem. Int. Ed. 2015, 54, 7349**
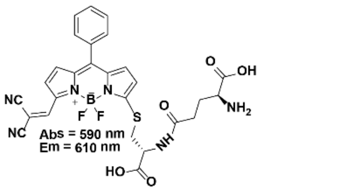	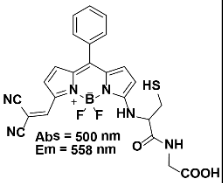	**Ratiometric** Ex = 514 nm	**18.69**	**N.D.**	**90**	**52**	**Biomaterials, 2018, 173, 1**
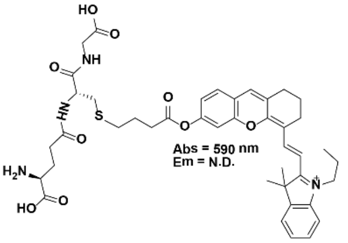	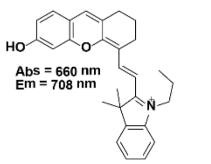	**Turn on** Ex = 680 nm	**7.01**	**500**	**70**	**N.D.**	**Biosen. Bioelectron. 2016, 81, 395**
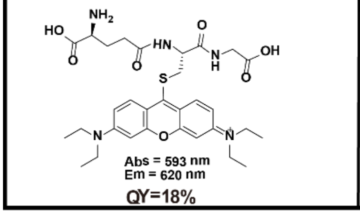	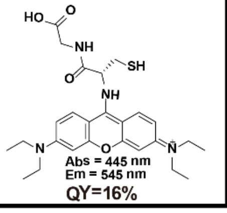	**Ratiometric** Ex = 488 nm	**22.6**	**10**	**147**	**75**	**This work**

Table S1. continued

NMR spectra of compounds

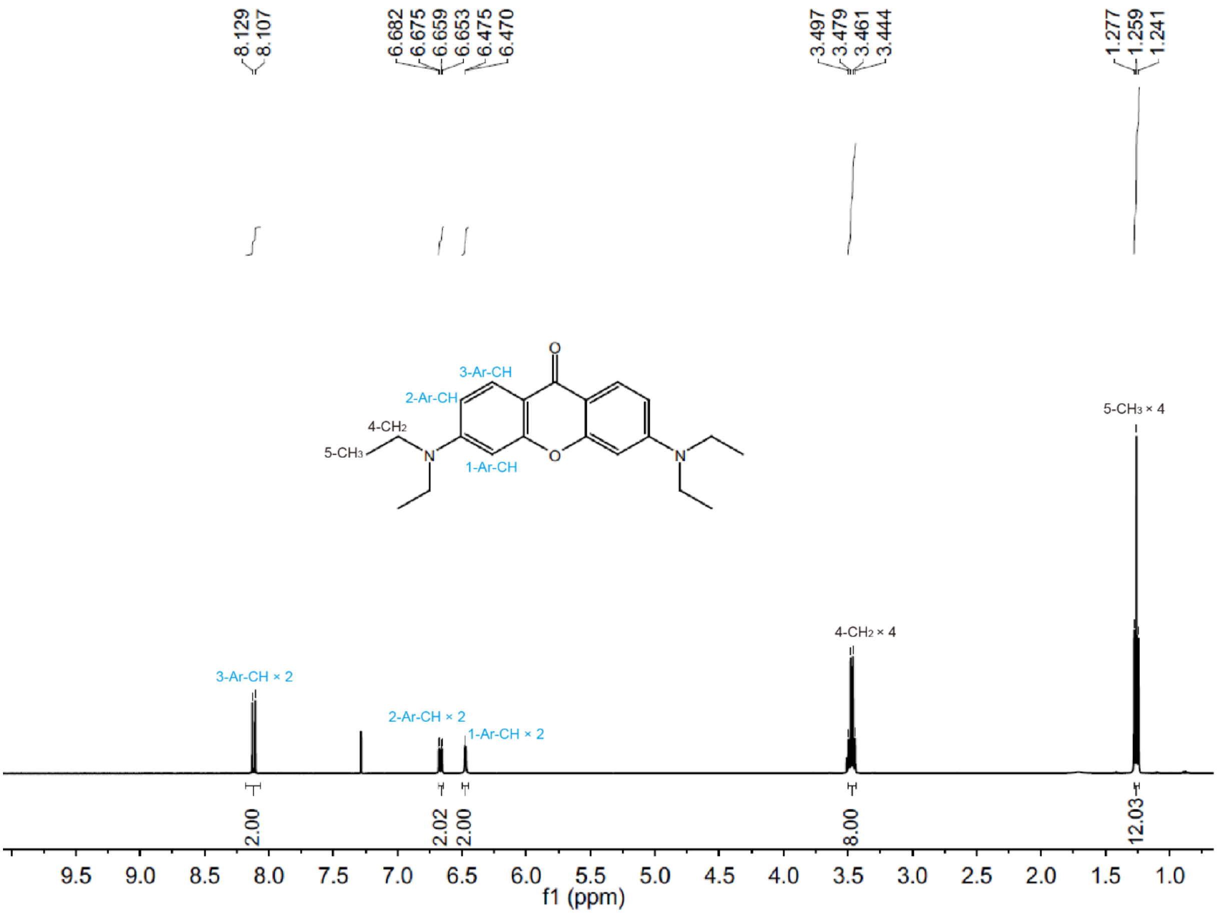

^{1}H NMR of compound S2

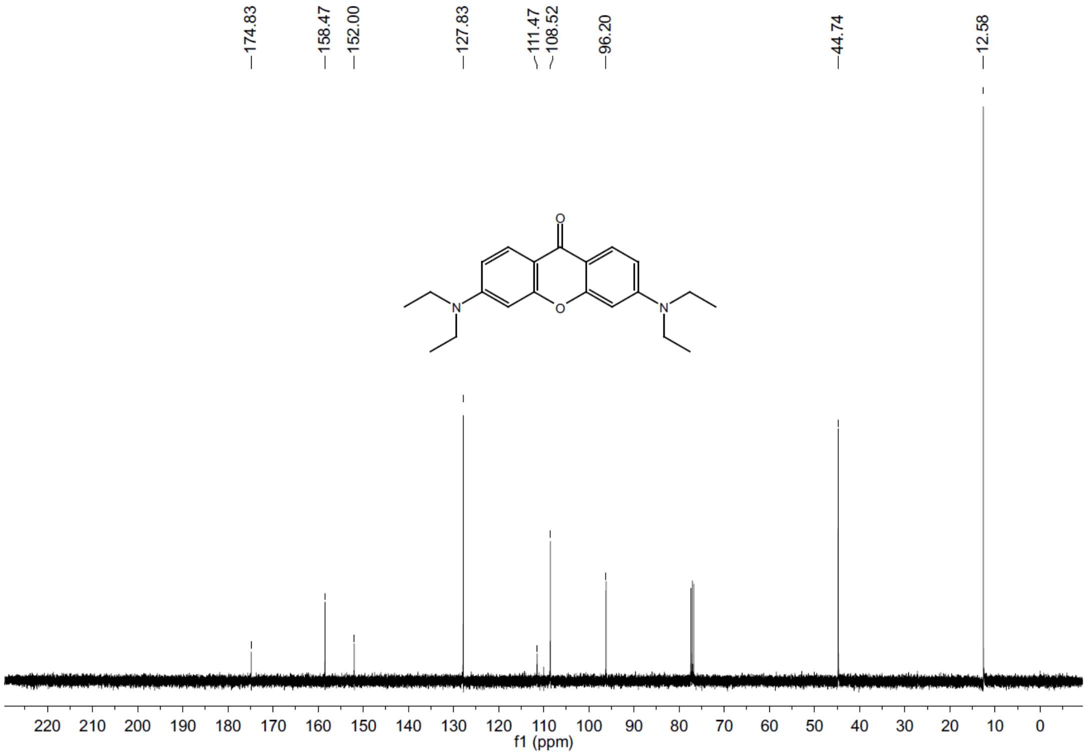

^{13}C NMR of compound S2

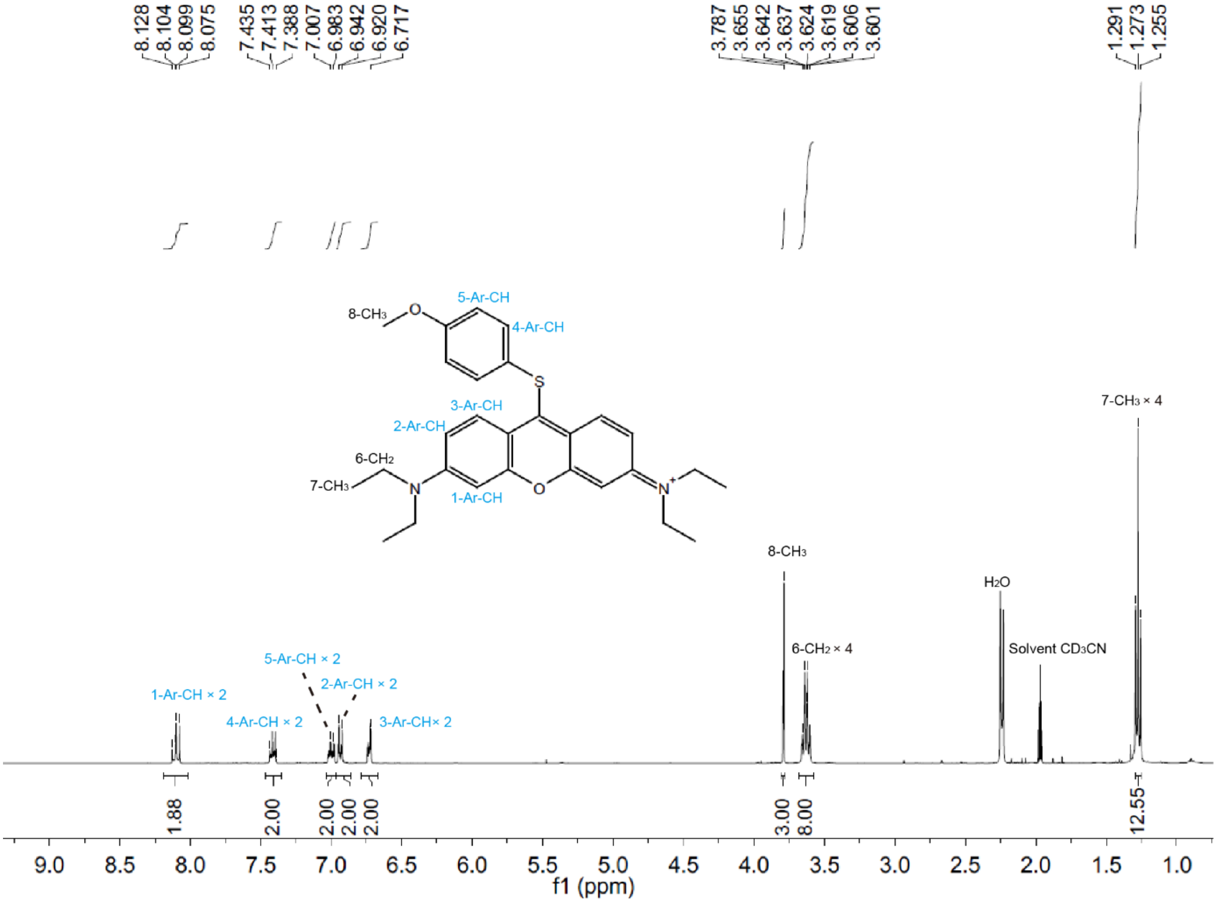

^{1}H NMR of compound S3

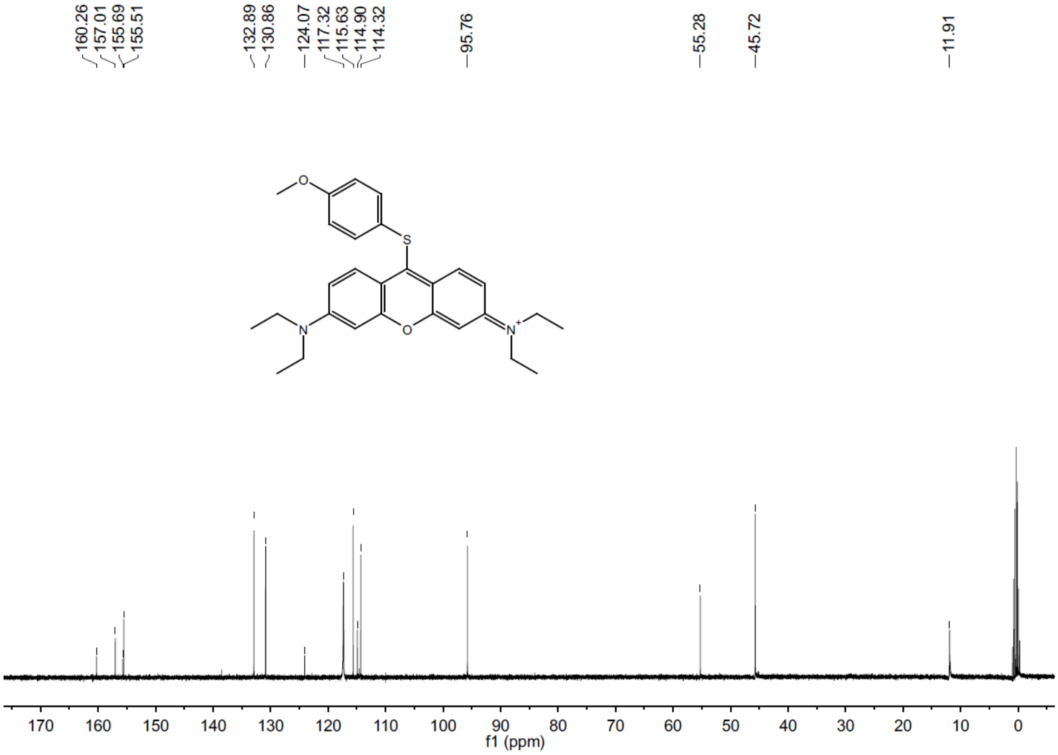

^{13}C NMR of compound S3

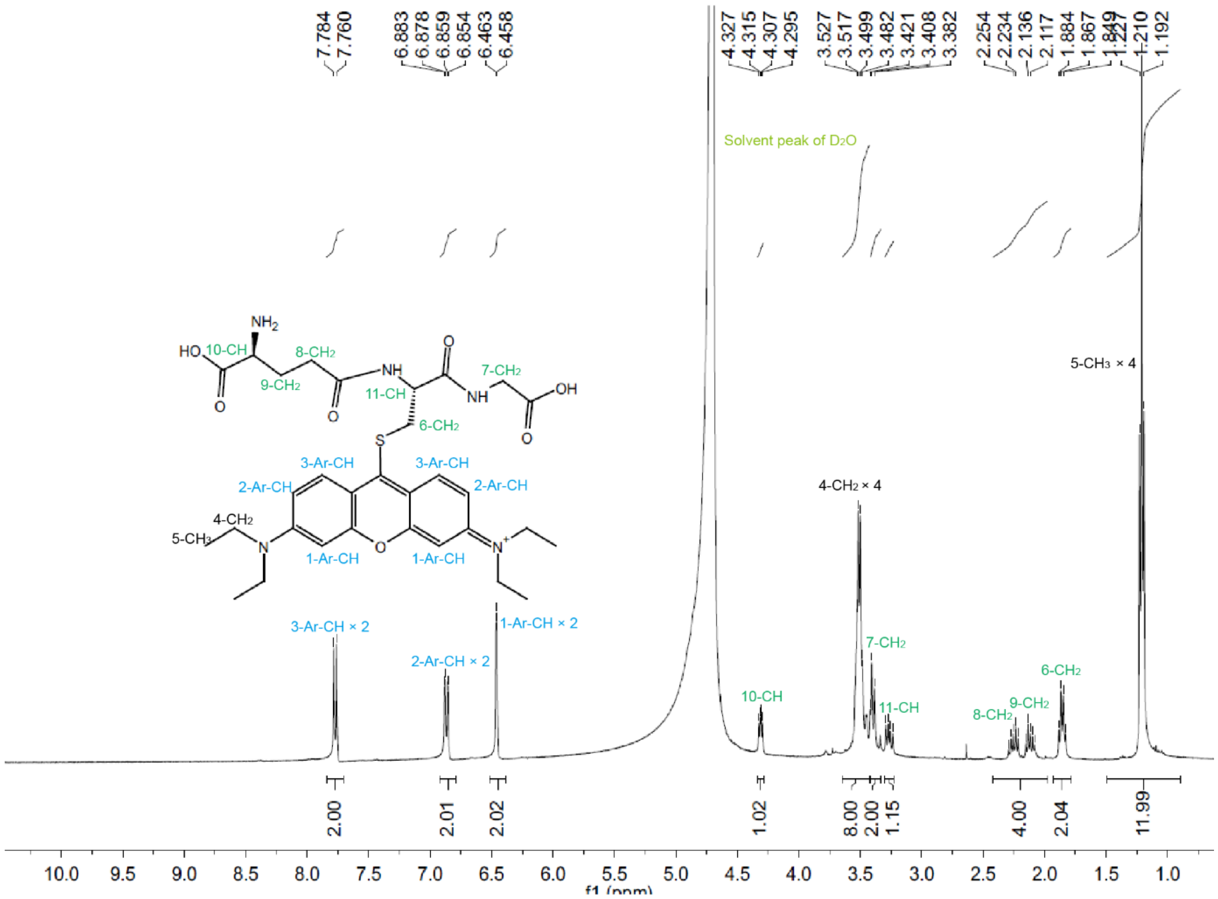

^{1}H NMR of Py-GSH

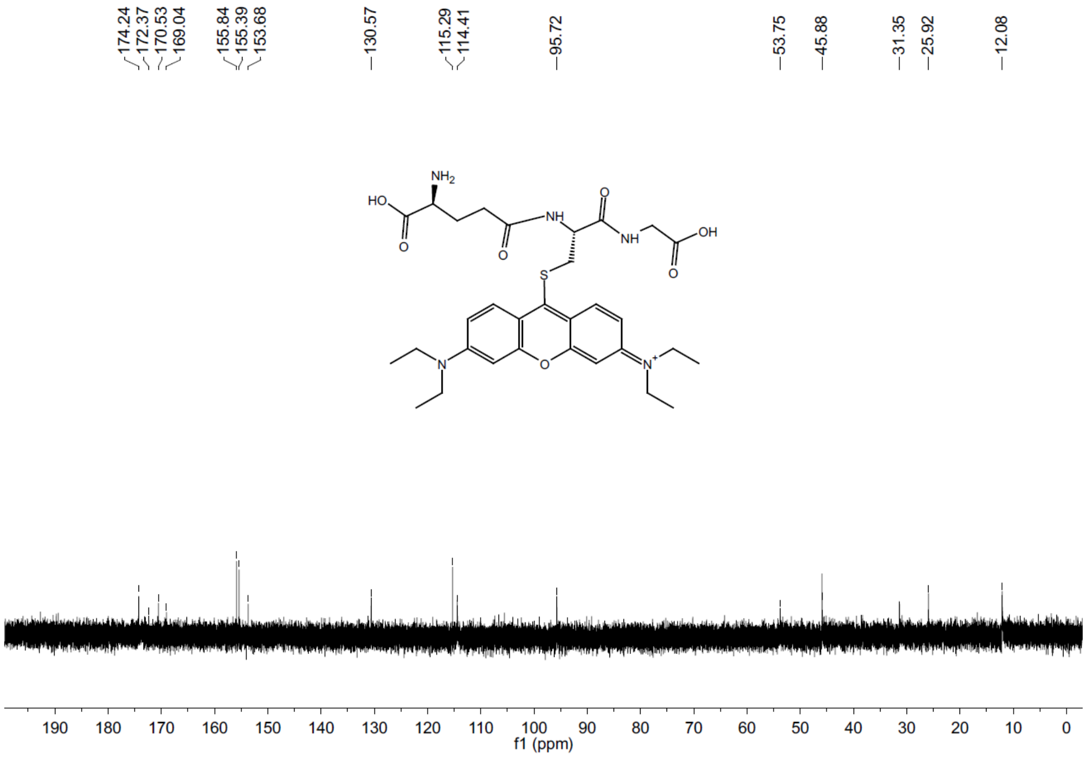

^{13}C NMR of compound Py-GSH

Supplemental Reference

1. P. Shieh, V. T. Dien, B. J. Beahm, J. M. Castellano, T. Wyss-Coray and C. R. Bertozzi, *J. Am. Chem. Soc.* **2015**, *137*, 7145.
2. J. Liu, Y. Q. Sun, H. X. Zhang, Y. Y. Huo, Y. W. Shi and W. Guo. *Chem. Sci.*, **2014**, 5, 3183.
3. M. J. Frisch, G. W. Trucks, H. B. Schlegel, G. E. Scuseria, M. A. Robb, J. R. Cheeseman, J. A. Montgomery, Jr., T. Vreven, K. N. Kudin, J. C. Burant, J. M. Millam, S. S. Iyengar, J. Tomasi, V. Barone, B. Mennucci, M. Cossi, G. Scalmani, N. Rega, G. A. Petersson, H. Nakatsuji, M. Hada, M. Ehara, K. Toyota, R. Fukuda, J. Hasegawa, M. Ishida, T. Nakajima, Y. Honda, O. Kitao, H. Nakai, M. Klene, X. Li, J. E. Knox, H. P. Hratchian, J. B. Cross, V. Bakken, C. Adamo, J. Jaramillo, R. Gomperts, R. E. Stratmann, O. Yazyev, A. J. Austin, R. Cammi, C. Pomelli, J. Ochterski, P. Y. Ayala, K. Morokuma, G. A. Voth, P. Salvador, J. J. Dannenberg, V. G. Zakrzewski, S. Dapprich, A. D. Daniels, M. C. Strain, O. Farkas, D. K. Malick, A. D. Rabuck, K. Raghavachari, J. B. Foresman, J. V. Ortiz, Q. Cui, A. G. Baboul, S. Clifford, J. Cioslowski, B. B. Stefanov, G. Liu, A. Liashenko, P. Piskorz, I. Komaromi, R. L. Martin, D. J. Fox, T. Keith,M. A. Al-Laham, C. Y. Peng, A. Nanayakkara, M. Challacombe, P. M. W. Gill, B. G. Johnson, W. Chen, M. W. Wong, C. Gonzalez and J. A. Pople, *GAUSSIAN 03 (Revision C.02)*, Gaussian, Inc., Wallingford, CT, 2004.
